# Novel multifunctional NIR-II aggregation-induced emission nanoparticles-assisted intraoperative identification and elimination of residual tumor

**DOI:** 10.1186/s12951-022-01325-9

**Published:** 2022-03-19

**Authors:** Qiaojun Qu, Zeyu Zhang, Xiaoyong Guo, Junying Yang, Caiguang Cao, Changjian Li, Hui Zhang, Pengfei Xu, Zhenhua Hu, Jie Tian

**Affiliations:** 1 College of Medical Imaging, Shanxi Medical University, Taiyuan, 030001 China; 2 CAS Key Laboratory of Molecular Imaging, Beijing Key Laboratory of Molecular Imaging, The State Key Laboratory of Management and Control for Complex Systems, Institute of Automation, Chinese Academy of Sciences, Beijing, 100190 China; 3 Beijing Advanced Innovation Center for Big Data-Based Precision Medicine, School of Medicine and Engineering, Beihang University, Beijing, 100191 China; 4 Department of Gastroenterology, The Third Medical Centre, Chinese PLA General Hospital, Beijing, 100190 China; 5 Anhui Medical University, Hefei, 230000 China; 6 Department of Hepatobiliary Surgery, Zhujiang Hospital, Southern Medical University, Guangzhou, 510280 China; 7 School of Artificial Intelligence, University of Chinese Academy of Sciences, Beijing, 100049 China; 8 Department of Radiology, First Hospital of Shanxi Medical University, Taiyuan, 030001 China; 9 Institute of Clinical Pharmacy & Pharmacology, Jining First People’s Hospital, Jining Medical University, Jining, 272000 China

**Keywords:** Aggregation-induced emission, NIR-II imaging, Phototherapy, Phototheranostic, Residual tumor

## Abstract

Incomplete tumor resection is the direct cause of the tumor recurrence and metastasis after surgery. Intraoperative accurate detection and elimination of microscopic residual cancer improve surgery outcomes. In this study, a powerful D1–π–A–D2–R type phototheranostic based on aggregation-induced emission (AIE)-active the second near-infrared window (NIR-II) fluorophore is designed and constructed. The prepared theranostic agent, A1 nanoparticles (NPs), simultaneously shows high absolute quantum yield (1.23%), excellent photothermal conversion efficiency (55.3%), high molar absorption coefficient and moderate singlet oxygen generation performance. In vivo experiments indicate that NIR-II fluorescence imaging of A1 NPs precisely detect microscopic residual tumor (2 mm in diameter) in the tumor bed and metastatic lymph nodes. More notably, a novel integrated strategy that achieves complete tumor eradication (no local recurrence and metastasis after surgery) is proposed. In summary, A1 NPs possess superior imaging and treatment performance, and can detect and eliminate residual tumor lesions intraoperatively. This work provides a promising technique for future clinical applications achieving improved surgical outcomes.

**Graphic Abstract:**

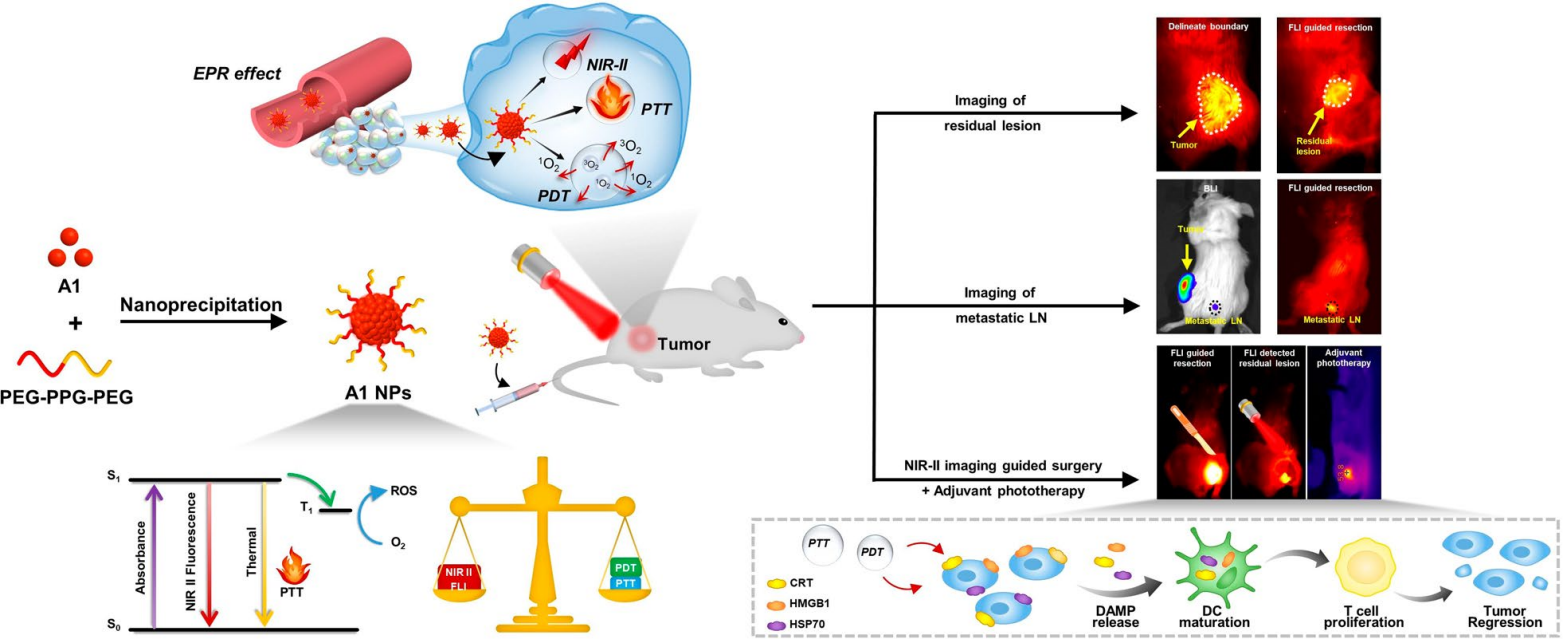

**Supplementary Information:**

The online version contains supplementary material available at 10.1186/s12951-022-01325-9.

## Introduction

Incomplete tumor resection is a prevalent cause of the recurrence and metastasis after surgery [1–3]. Statistically, incomplete tumor resection occurs in 20% breast cancer, 28% colorectal cancer and 30% head and neck cancer, which has become a significant clinical problem [4–6]. Currently, intraoperative assessment about whether the tumor has been completely removed is mainly dependent on surgeons’ visual inspection and palpation. However, identifying residual disease based on these subjective parameters is challenging [7]. Therefore, development of an intraoperative real-time imaging technique to accurately identify microscopic residual lesion is urgent needed.

Near-infrared (NIR) fluorescence imaging techniques provide new opportunities to address the above issues because of their high sensitivity, high resolution and real-time imaging capability [8, 9]. The clinical trials on NIR-I (700–900 nm) fluorescence imaging of OLT38, LUM015 and Bevacizumab-IRDye800CW have achieved rapid identification of residual tumor and lymph nodes during surgery for patients and may thus improve surgical outcomes [10–12]. Along with imaging technological developments, the second near-infrared window (NIR-II, 1000–1700 nm) fluorescence imaging has been developed, which has shown the far superior imaging quality than traditional NIR-I fluorescence imaging because of high imaging resolution, low background and autofluorescence, deep tissue imaging capability, etc [13–15]. Our previous study about the liver-tumor surgery guided by indocyanine green (ICG) fluorescence imaging demonstrated that comparing with NIR-I imaging, NIR-II imaging provides a higher minor tumor detection sensitivity. However, the maximum emission wavelength of ICG is at 845 nm and its quantum yield in the NIR-II window is relatively low. Therefore, there is an urgent need to develop applicable NIR-II fluorophores with both good biocompatibility and high quantum yield [16].

In many clinical practices, complete tumor resection is difficult and debulking surgery is usually used to relief the patient’s symptoms for some advanced tumors such as ovarian carcinoma, neoplasms of central nervous system and lymphoma [17]. In order to prolong the patients’ lifetimes, efforts have been devoted to explore new intraoperative adjuvant techniques to impede the residual tumour growth, such as intraoperative radiotherapy [18]. However, intraoperative radiotherapy can induce ionizing radiation damage. Recently, phototherapy including photothermal therapy (PTT) and photodynamic therapy (PDT) has attracted lots of attentions as a promising cancer treatment strategy for their feature of noninvasive and high selectivity [19, 20]. More importantly, PTT has been reported to supplement surgery and eliminate tiny residual tumor intra-operatively and achieved a better outcome [21, 22].

Aiming to reduce incomplete tumor resection rate and improve surgical outcome, we developed a multifunctional phototheranostics though a novel molecular design strategy in this study. The key structural feature of D1–π–A–D2–R type fluorophore A1 is the integration of planar π-conjugated units and twisted motifs into one molecule: (1) The planar motif has good conjugation and can increase the absorptivity; (2) the twisted unit can produce a higher ΦPL in aggregate because of the restriction of intermolecular interactions; (3) intramolecular motions are still active due to their loose packing in aggregate state. A1 NPs simultaneously shows high absolute quantum yield (1.23%), excellent photothermal conversion efficiency (55.3%), high molar absorption coefficient and moderate singlet oxygen generation performance. In addition, it was found that NIR-II fluorescence imaging of A1 NPs accurately detected residual tumor and metastatic lymph nodes in tumor-bearing mice models. Most importantly, we proposed an integrated strategy that NIR-II fluorescence-guided primary tumor resection and subsequent intraoperative fluorescence-guided phototherapy elimination of unresectable residual tumors to further improve surgical margins. In summary, this work provided new insight for effectively reducing the local recurrence and reoperation rate after surgery.

## Results

### Design, synthesis and characterization of the A1 molecule

**Fig. 1:**
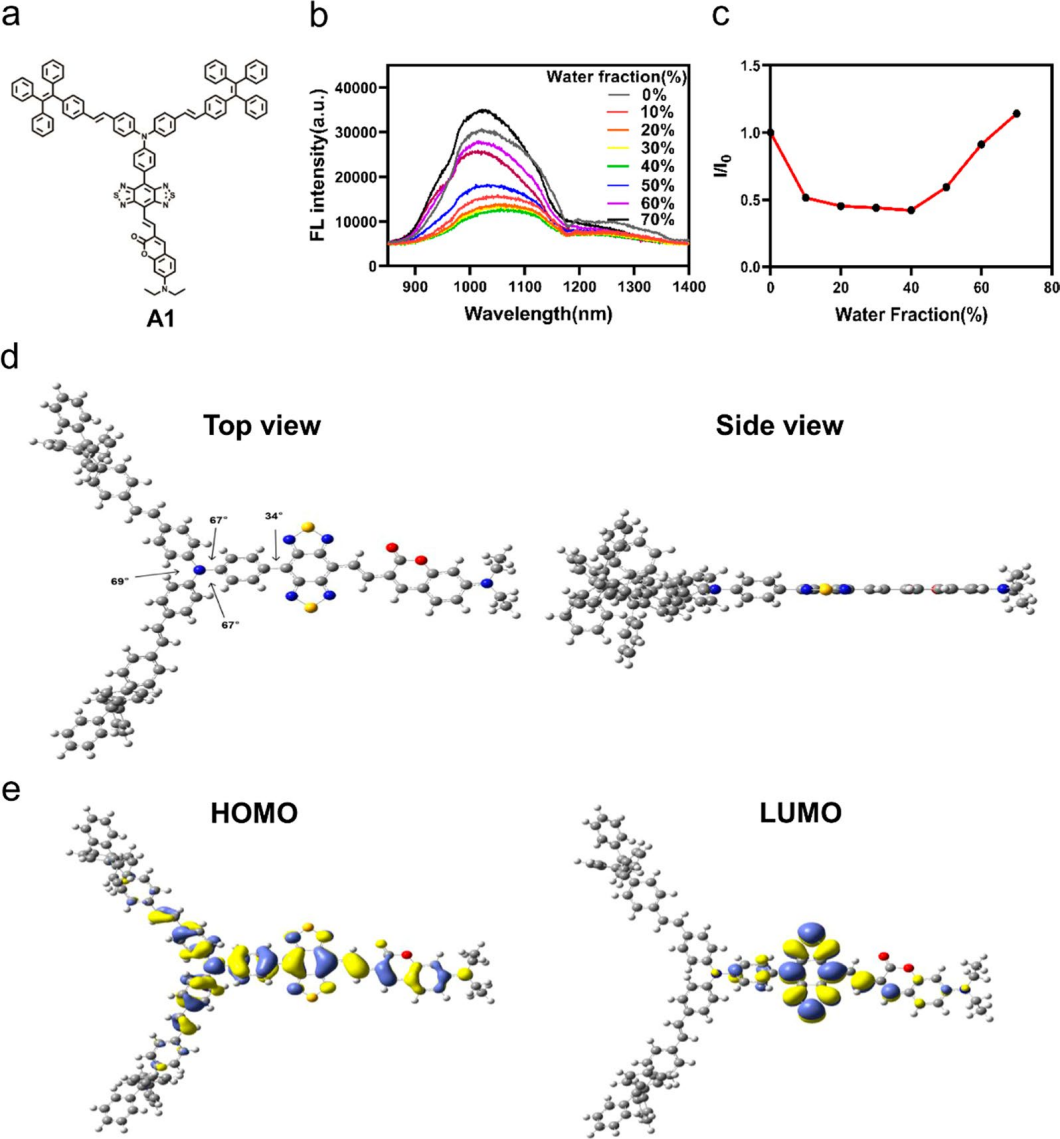
**a** Chemical structure of A1. **b** FL spectrum of A1 in THF/water mixture different f_w_. The concentration of A1 in the mixture is 7 nM. **c** I/I_0_ of A1 in THF/water mixture with different water fractions. **d** Optimized confirmation of A1 structure from top-view and side-view respectively. **e** HOMO and LUMO wave functions in the geometrically optimized structures

The excitation and emission of luminogens are strongly linked to band gap between the highest occupied molecular orbital (HOMO) and the lowest unoccupied molecular orbital (LUMO) of the ground state and excited state, respectively. So far, most NIR-II AIEgens are designed adopting the donor–acceptor–donor (D–A–D) structure, which reduces the band gaps by enhancing the D–A effect. Benzo[1,2-c:4,5-c′]bis([1,2,5]thiadiazole) (BBTD) is an extremely electron-deficient unit which has been widely used as an important part of the NIR-II emitter. Cheng’s group designed a new type of NIR II organic fluorophore CH1055 composed of the BBTD acceptor and triphenyl amine donor with the dihedral angles between donor and acceptor units over 30° recently. This torsional distortion reduces the delocalization of donor-acceptor π-electrons due to poor overlap of orbital interactions, resulting in a low absorption coefficient [14]. As reported before, the molar absorption is sensitive to the conformation of the central π system. If a π system acquires a planar conformation, the electronic coupling can be optimized and would maximize the π-orbital overlap and oscillator strength. As a coplanar fluorophore, coumarin dyes have good photochemical properties such as large fluorescence quantum yields, high molar extinction coefficients, and tolerance to photobleaching. In this work, we proposed a kind of D1–π–A–D2–R type fluorophore termed A1 (Fig. 1a). Fluorophore A1 is comprised of 7-(Diethylamino)coumarin (Donor 1), triphenylamine segment (Donor 2), Benzo[1,2-c:4,5-c′]bis([1,2,5]thiadiazole) (BBTD, Acceptor) and tetraphenylethene (TPE, molecular rotor), in which the good conjugation enables strong absorption and the molecular rotation and vibration afford AIE signature. This fluorophore can be fine-tuned to achieve bright emission at NIR-II region with good absorption coefficient while subtly balancing the AIE effect. In addition, in the aggregated state, the highly distorted conformation of the TPE segment will lead to relatively loose accumulation of molecules, extending the intermolecular distance, helping to retain part of the intramolecular rotation, and thus conducive to non-radiative energy dissipation (e.g., nonradiative decay and ROS generation). As for the ROS generation, it attributes to the process of forming AIE aggregates of fluorophore A1 can improve the energy match between excited singlet and triplet states, thereby reducing their energy gap (ΔEST). Consequently, the yield of the triplet excited state would be improved thanks to the promoted intersystem crossing (ISC) rate, hence boosting _1_O^2^ generation.

The synthetic route of A1 is showed in Additional file 1: Scheme S1. Briefly, compound 1 and compound 3 were constructed through the previous method [23]. Compound 2 was synthesized by two-step stannylation/Stille cross-coupling protocol from compound 1. The stannylation of compound 3 with 1,1,1,2,2,2-hexabutyldistannane afforded Compound 4, which was then reacted with Compound 2 at the catalyst of Pd(PPh3)4 affording compound 5. Finally, A1 was obtained in 67% yield through Wittig reaction from compound 5 and 4,4′-((4-bromophenyl)azanediyl) dibenzaldehyde.

**Fig. 2:**
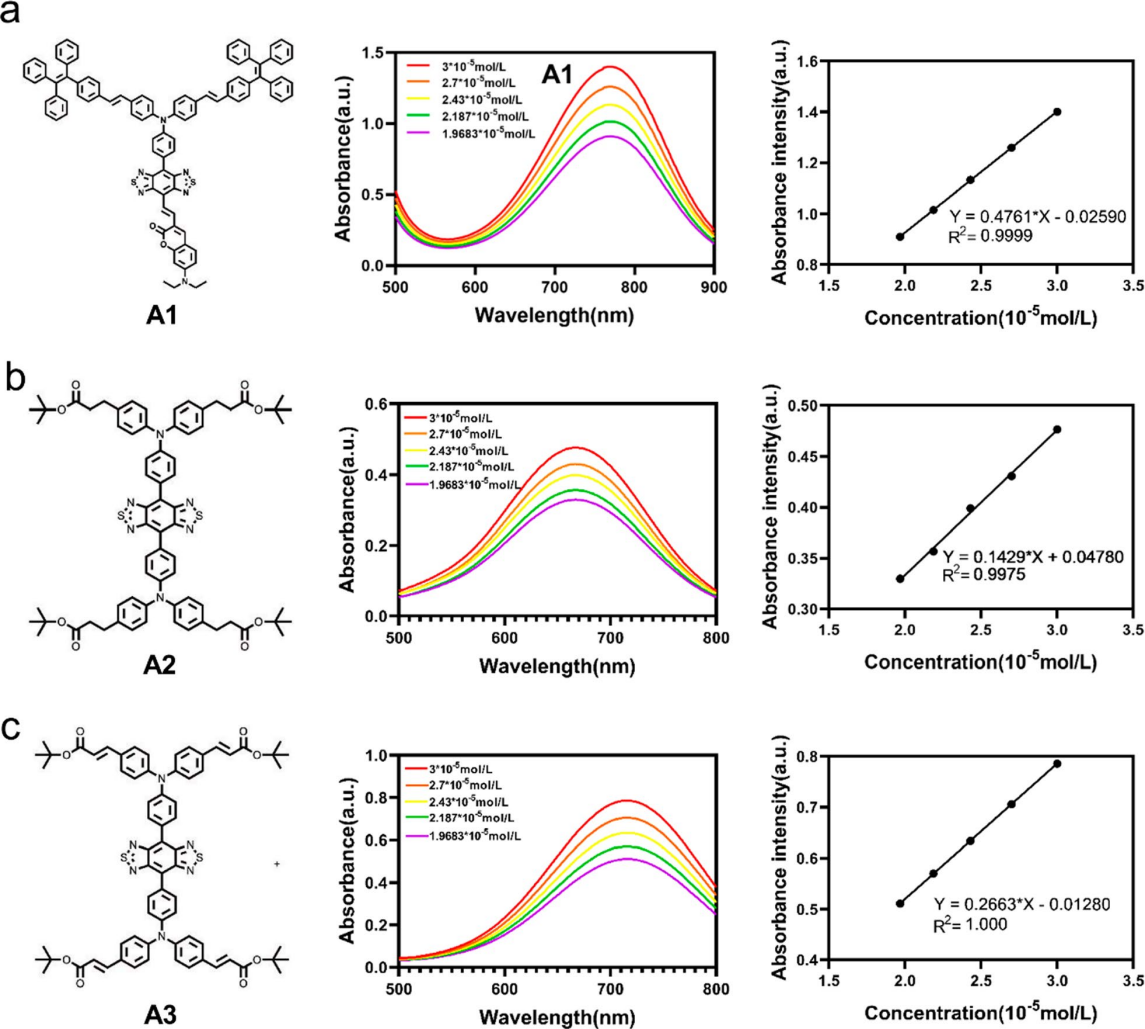
**a** Schematic chemical structure of A1, Absorption curves of A1 in THF at different concentrations and corresponding Linear absorbance versus concentration; **b** Schematic chemical structure of A2, Absorption curves of A2 in THF at different concentrations and corresponding Linear absorbance versus concentration; **c** Schematic chemical structure of A3, Absorption curves of A3 in THF at different concentrations and corresponding Linear absorbance versus concentration

To investigate the geometric and electronic properties of A1, we carried out density functional theory calculation. As shown in Fig. 1d, the dihedral angle between BBTD plane and triphenylamine segment was calculated to be 34°. Furthermore, the TPEPY moieties were oriented out of the plane of the conjugated backbone. These twisted conformations of A1 would prevent π–π stacking in aggregate state. There is a vinyl bridge between BBTD plane and coumarin group, it leads to a nearly coplanar conformation endowing A1 with a significantly enhanced ε of 4.761 * 10^− 4^ M^− 1^ cm^− 1^ compared with A2 (ε = 1.429 * 10^− 4^ M^− 1^ cm^− 1^) and A3 (ε = 2.663 * 10^− 4^ M^− 1^ cm^− 1^) (Fig. 2a–c). In terms of the electronic structures, the HOMO wave functions are well delocalized along the whole molecule backbone, while the LUMO wave functions are mainly localized on the D1–π–A–D2 core with good conjugation (Fig. 1e). In addition, the AIE property of A1 was investigated in THF/water mixtures with different water fractions (f_w_). The results showed that the fluorescence intensity gradually decreased with an increase in f_w_ from 0 to 30%, increased when the f_w_ exceeded 40%. Besides, A1 showed a stronger NIR-II fluorescence signal at 70% f_w_ than at 0% f_w_ (Fig. 1b and c), indicating the typical AIE property of A1.

### Design, synthesis and characterization of the A1 NPs

**Fig. 3:**
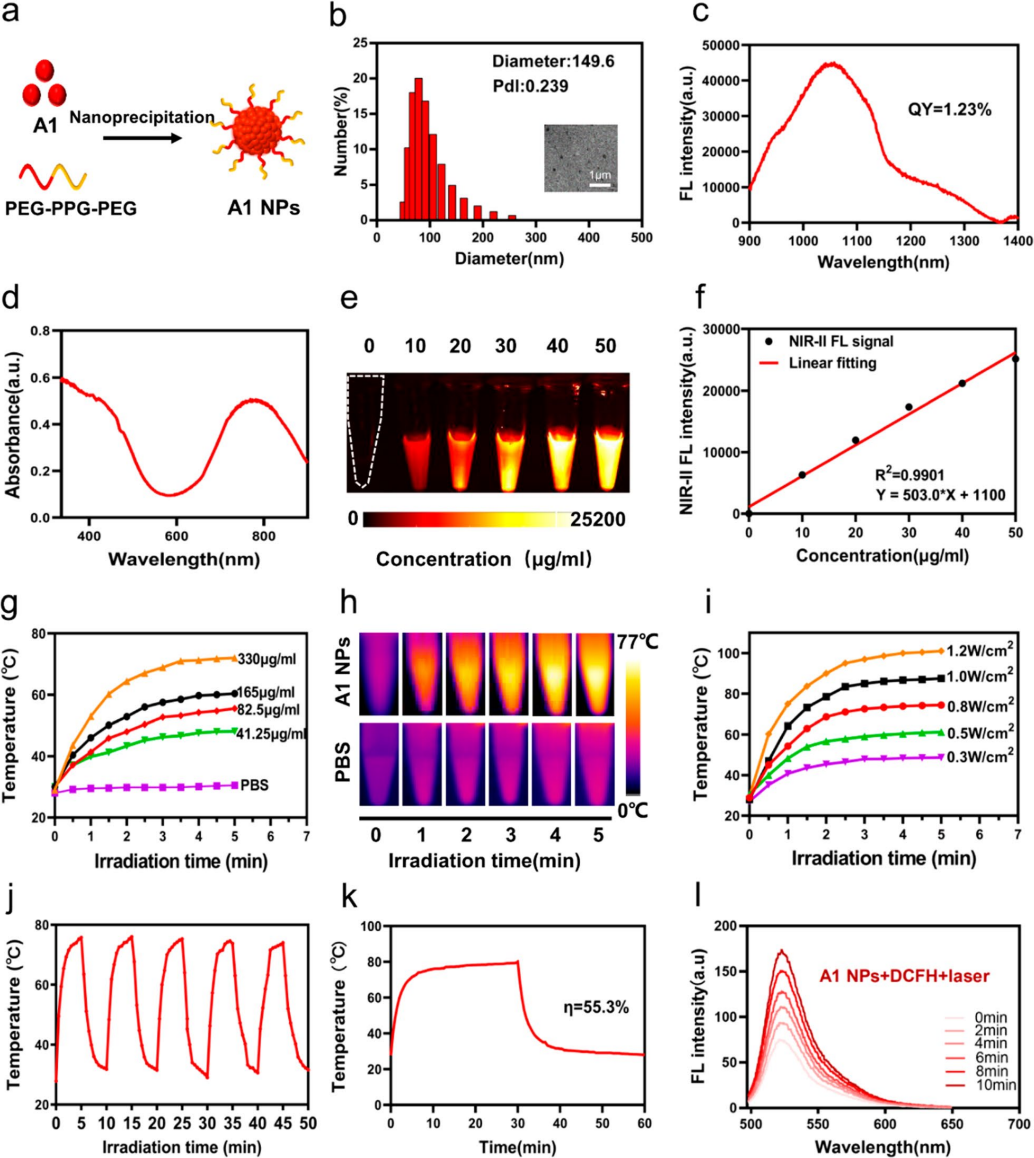
**a** Preparation of A1 NPs. **b** DLS data and TEM image of A1 NPs. **c**, **d** emission spectra and absorption spectra of A1 NPs in water. **e** Fluorescent images of different concentrations A1 NPs aqueous solution under 808 nm laser irradiation. **f** Function graph of fluorescence signal intensity at different concentrations. **g** The heating curve of different concentrations of A1 NPs under 808 nm irradiation (0.8 W/cm^2^). **h** Photothermal images of A1 NPs aqueous and PBS (330 µg/ml) after 5 min of 0.8 W/cm^2^ 808 nm laser irradiation. **i** Photothermal heating curves of aqueous dispersions of A1 NPs (330 µg/ml) under different laser power. **j** Temperature change of A1 NPs aqueous solution over five on/off cycles of 808 nm laser irradiation (0.8 W/cm^2^, 330 µg/ml). **k** Temperature-increasing/decreasing curve. **l** Fluorescence spectra of DCFH under 808 nm laser irradiation (0.1 W/cm^2^) in the presence of A1 NPs at different post-irradiation times (0, 2, 4, 6, 8, and 10 min)

A1 molecule showed poor water-soluble and was not conducive to biological applications, thus A1 nanoparticles (NPs) were prepared using a nanoprecipitation method (Fig. 3a). Dynamic light scattering (DLS) was used to determine A1 NPs size. As shown in Fig. 3b, A1 NPs’ average hydrodynamic diameter was 149.6 nm. The A1 NPs exhibited fluorescence signal from 900 to 1200 nm and the peak emission is at 1050 nm (Fig. 3c). NIR-II quantum yield (QY) of A1 NPs was calculated to be 1.23%. Subsequently, we obtained fluorescence signal intensity of different concentrations of A1 NPs (Fig. 3e). Specifically, the correlation between NIR-II imaging intensity and A1 NPs concentration was found to be Y = 503.0 × X + 1100 (R^2^ = 0.99) (Fig. 3f). We then studied the photothermal performance of the A1 NPs. The A1 NPs showed outstanding photothermal conversion, and the temperature increased with the concentration and laser power increased (Fig. 3g–i). No significant change of photothermal performance after five times ON/OFF laser cycles was observed, showing the high photothermal stability of the A1 NPs (Fig. 3j). The photothermal conversion efficiency of the A1 NPs was calculated to be 55.3% (Fig. 3k). The photodynamic effect of A1 was also evaluated. It was seen that the fluorescence intensity of 2′,7′-Dichlorofluorescein (DCFH) containing A1 NPs aqueous dispersion increased obviously post irradiation, indicating the A1 NPs have photodynamic effect (Fig. 3l). In addition, we monitored A1 NPs stability (Additional file 1: Fig. S12). The above results demonstrated that A1 NPs had attractive optical and treatment properties in vitro.

### NIR-II fluorescence imaging and biodistribution of A1 NPs in vivo

**Fig. 4:**
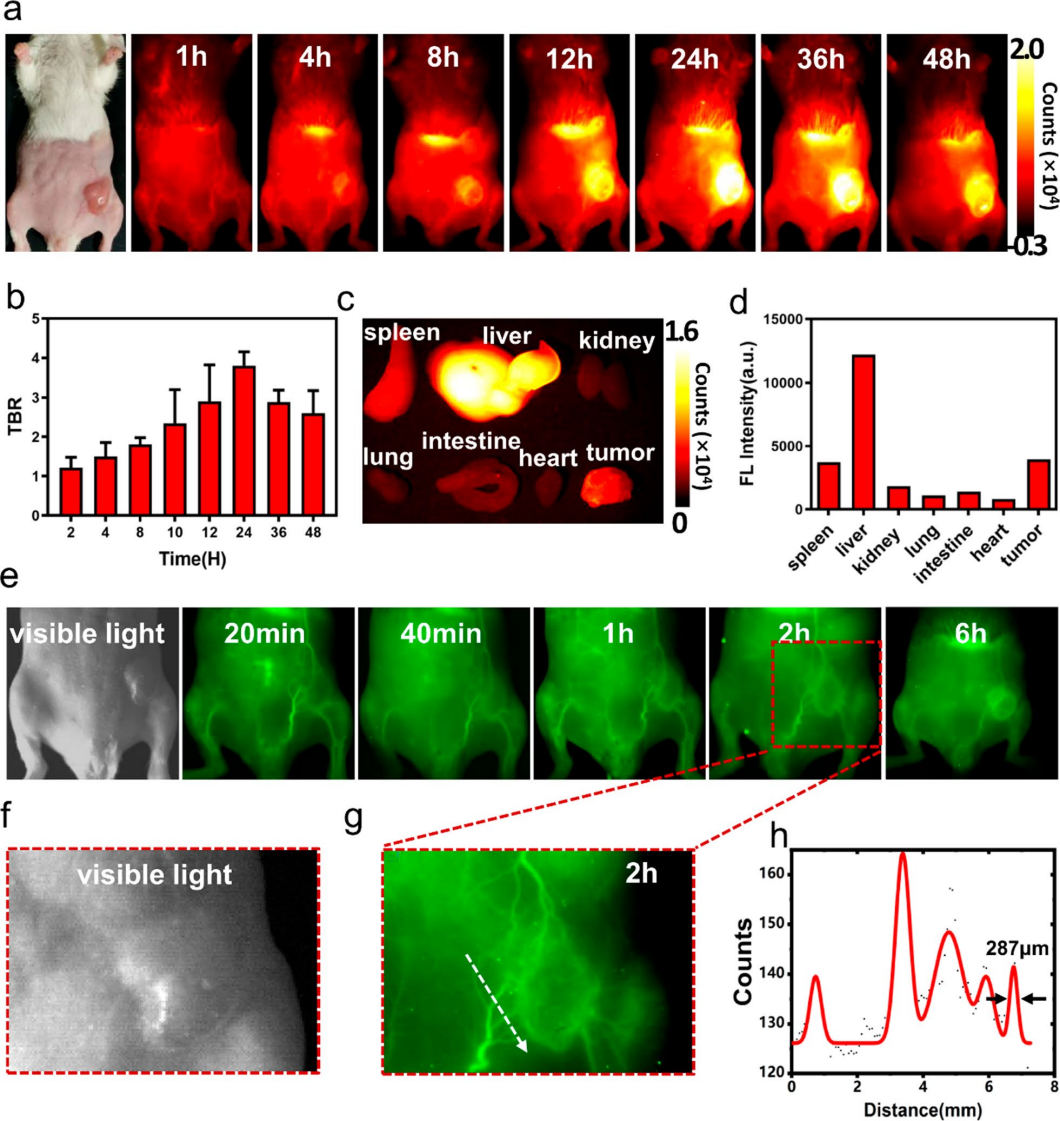
**a** Representative NIR-II fluorescence images at various time points after tail-vein administration of A1 NPs. **b** Tumor TBR of different time points after tail-vein injection of A1 NPs. **c**, **d** NIR-II images and fluorescence signal intensity of the tumor and main organs at 108 h after A1 NPs injection. **e**–**g** Tumor and adjacent vascular imaging at different time points of tumor-bearing mice. **h** Cross-sectional fluorescence intensity profiles of white arrows as indicated in **g**

The NIR-II imaging efficacy of the A1 NPs in vivo was examined. After A1 NPs was injected via tail vein in 4T1 tumor-bearing mice, it was found that the A1 NPs specifically accumulated at the tumor site (Fig. 4a, Additional file 1: Fig. S13) and the tumor-to-background ratio (TBR) reached a maximum value (3.8 ± 0.35) at 24 h (Fig. 4b). Thus, 24 h post-injection of the A1 NPs was selected as the optimal imaging and phototherapy time point. Then, by using a homemade NIR-II fluorescence microscopy imaging system to image the tumor frozen sections, it was found that the probes accumulated in tumor tissues (Additional file 1: Fig. S14). Major organs were harvested at 108 h to study the biodistribution of the NPs, the results showed that the highest fluorescence signal in liver and no significantly fluorescence signal in lungs, kidney, heart and intestine (Fig. 4c and d). In addition, A1 NPs showed relatively long blood circulation times. Tumor vessels were clearly seen during 6 h post-injection. It was found that 4T1 breast tumors had a rich blood supply, and A1 NPs gradually distributed from tumor periphery to the tumor center after the injection (Fig. 4e–g). From high local magnification imaging of tumor, microvessel branches of tumor less than 300 μm in diameter were observed (Fig. 4h).

### NIR-II fluorescence imaging-guided residual tumors and metastatic lymph nodes resection in vivo

**Fig. 5:**
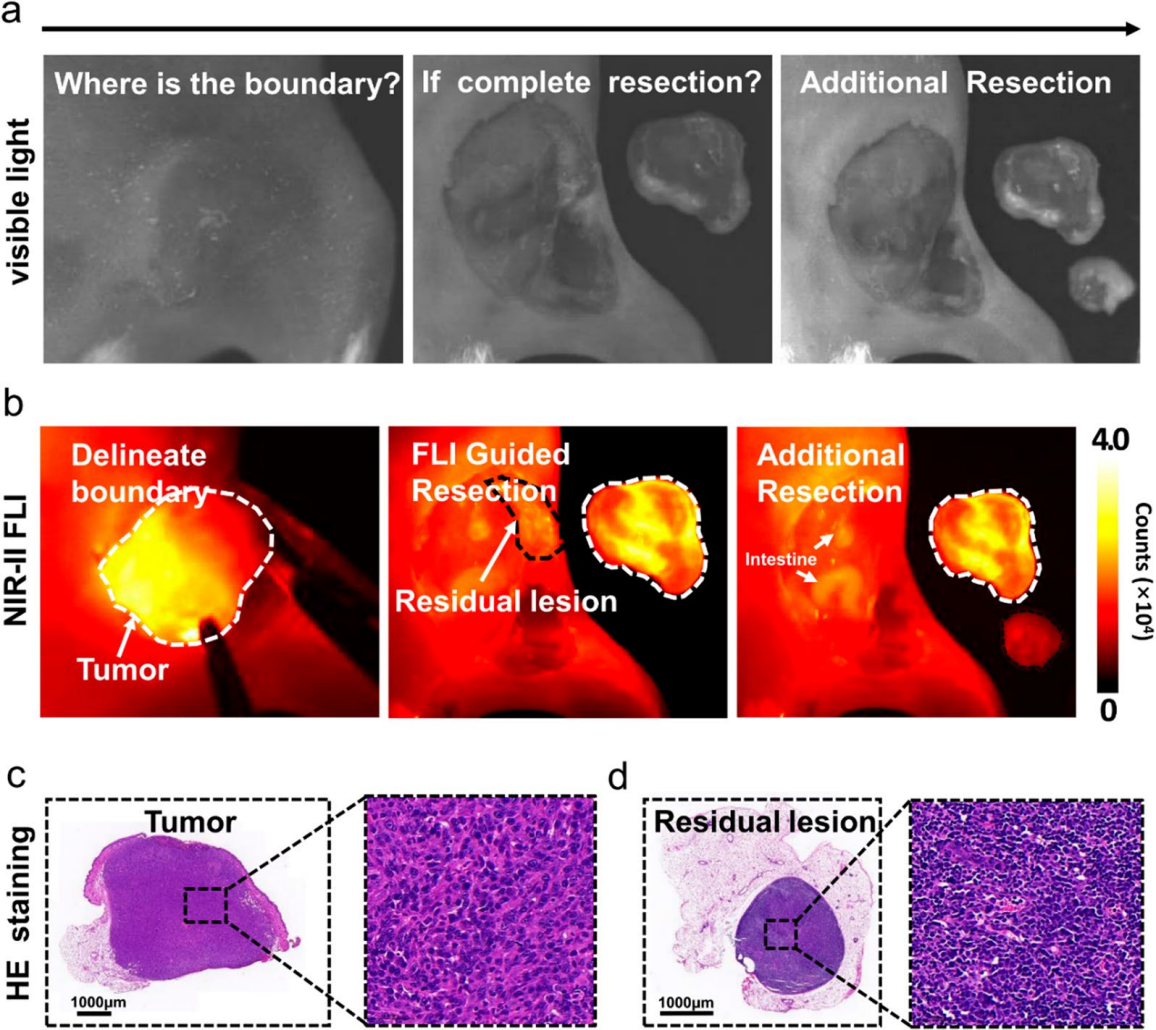
**a**, **b** Visible light images and corresponding NIR-II images of orthotopic 4T1 tumor resection procedure. **c**, **d** H&E staining images of excised tumor and residual lesion

Based on the finding that specific accumulation of A1 NPs at the tumor and good TBR, NIR-II fluorescence imaging-guided surgery was performed. Before the start of surgery, fluorescence imaging delineated tumor boundaries and assist surgeon to make operative planning. After the surgeon subjectively felt that he had completely removed the tumor, an 808 nm laser was again used to scan the tumor cavity. Residual fluorescent signal was detected in the operation area in 4 mice and then was resected (Fig. 5a and b, Additional file 1: Fig. S15a, d). H&E staining results of all resected lesion confirmed the presence of the tumor tissue (Fig. 5c and d, Additional file 1: Fig. S15b, c, e, f). The ratio of average fluorescence intensity from the residual tumors to that from surrounding normal tissues was 2.1. Owing to high signal-to-background ratio, microscopic tumor residues (2 mm in diameter) was clearly detected by the fluorescence imaging of the A1 NPs. All mice were cured without any local tumor recurrences during two months’ observation after surgery.

**Fig. 6:**
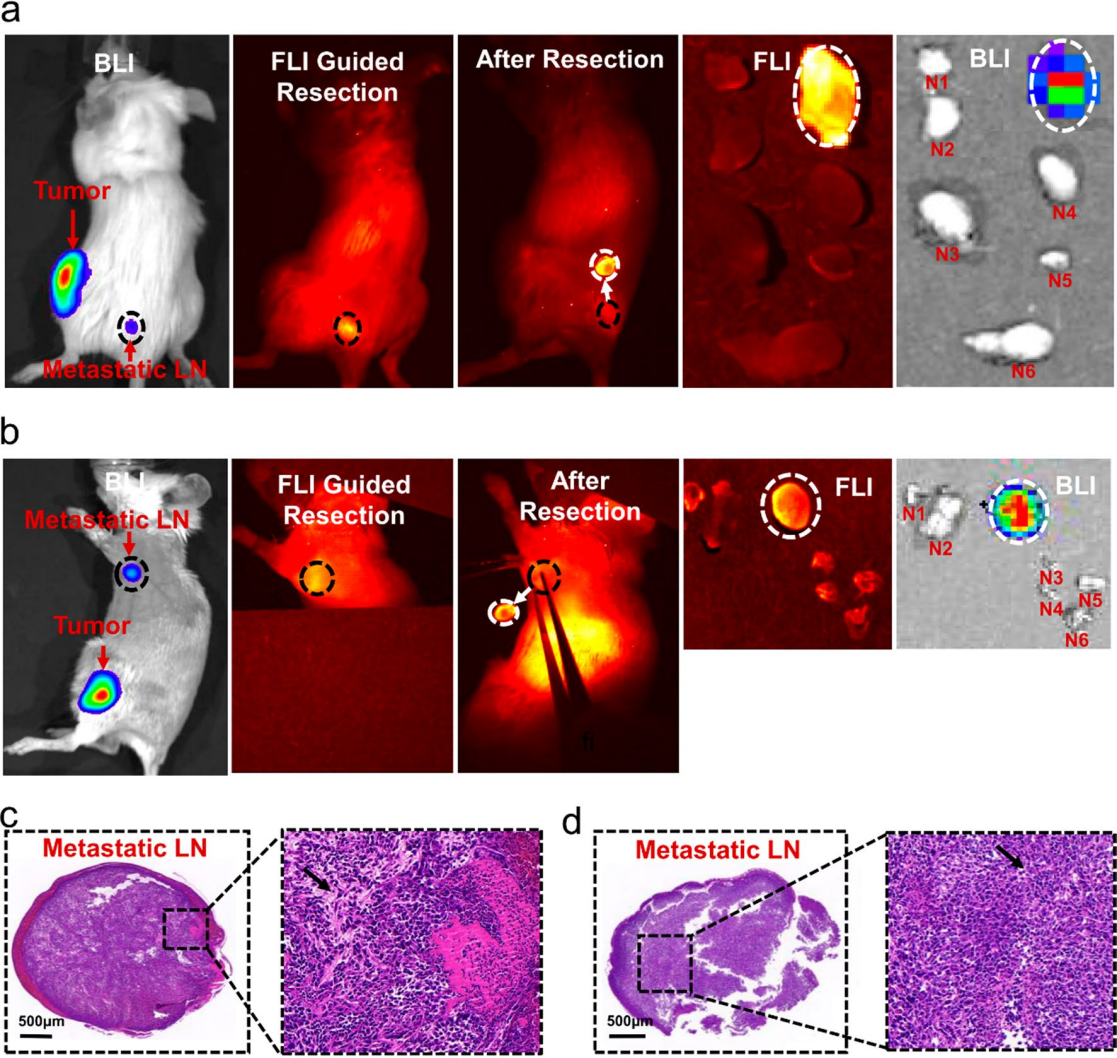
**a** BLI images detected a positive sciatic lymph node in orthotopic breast cancer mouse. A clear fluorescent signal matched well with that of the bioluminescence imaging (BLI). Positive lymph nodes were resected under the NIR-II fluorescence imaging. Afterwards, the mice were sacrificed, and the rest of the enlarged lymph nodes were harvested. All the ex vivo lymph nodes was performed immediately BLI and FLI. **b** An identical set of images about positive axillary lymph node. **c**, **d** H&E staining images of the positive sciatic lymph node and axillary lymph node

In this study, we also evaluated if NIR-II fluorescence imaging based A1 NPs could identify metastatic lymph nodes during operation. We successfully constructed positive lymph node models by monitoring bioluminescent imaging (BLI). Interestingly, a strong fluorescence signal was observed to match well with that of the BLI 24 h post A1 NPs intravenous injection. Lesion with strong fluorescence signal was removed and confirmed to be metastatic lymph nodes by pathology. In addition, the remaining enlarged lymph nodes were removed from the euthanized mice. When imaging these lymph nodes in vitro, it was found that they had only weak fluorescence and no bioluminescence signal, and finally they were confirmed to be non-metastatic lymph nodes by pathology (Fig. 6a–d, Additional file 1: Figs. S16–S18). Therefore, A1 NPs were likely to specifically label metastatic lymph nodes.

### Anticancer and inducing anti-tumor immune responses in vivo

**Fig. 7:**
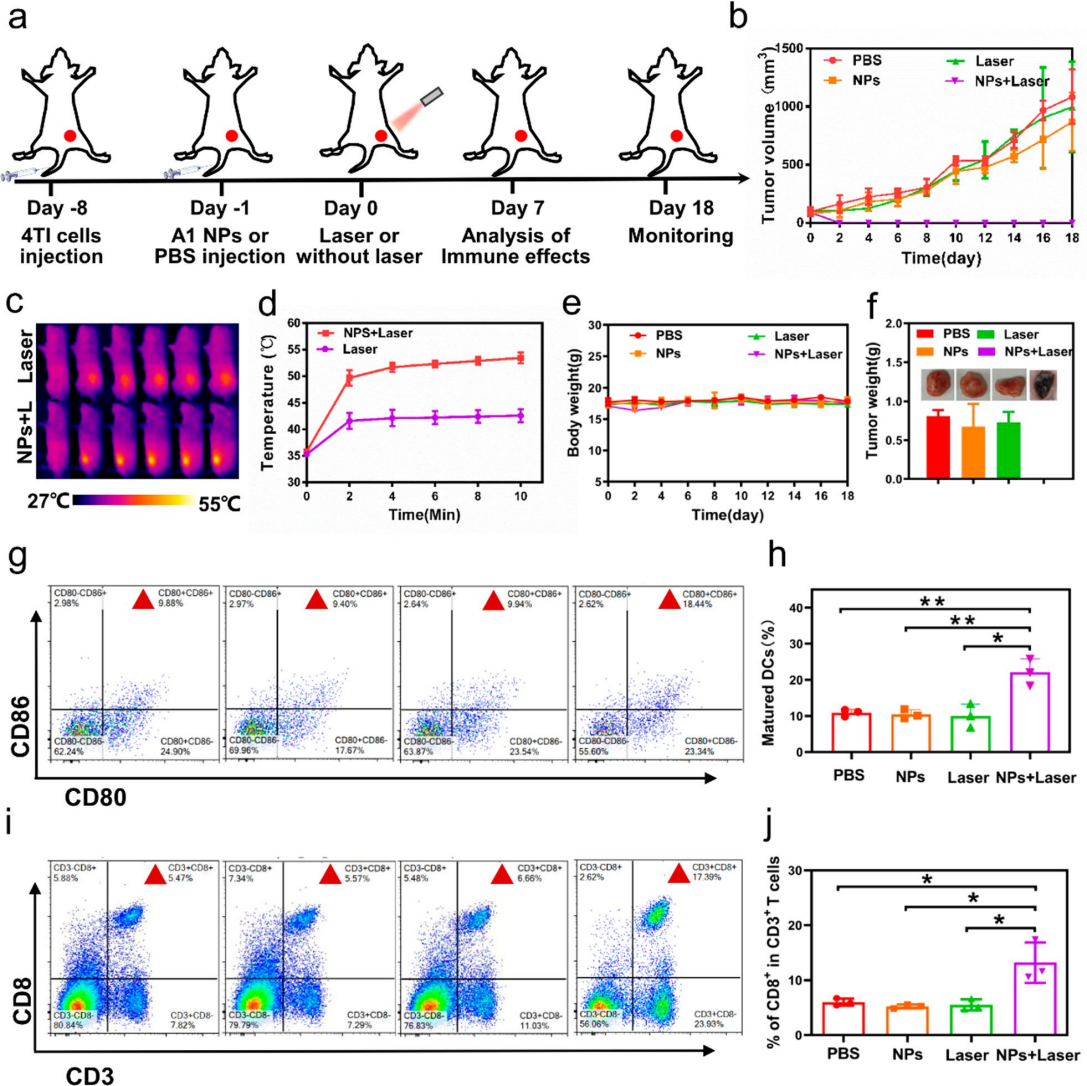
**a** The mice were received with the assigned schemes. **b** Tumor growth curve of four group mice after different treatment. **c** Photothermal images of 4T1 tumor-bearing mice in Laser group and A1 NPs + Laser group under laser irradiation for 10 min (808 nm, 1 W/cm^2^). **d** Tumor temperature curve at different time points of laser irradiation. **e** Changes in body weights of the mice among different groups. **f** Isolated tumor weights of different groups. **g**–**j** Flow cytometry analysis of DC maturation in tumor-draining lymph node (**g**, **h**) and CD3+ CD8+ lymphocytes (CTLs) in spleens (**i**, **j**) in different groups. (*P < 0.05, **P < 0.01)

The effects of synergistic phototherapy in anticancer and inducing anti-tumor immune responses were examined in vivo (Fig. 7a). As evident from the tumor volume curve, tumors were significantly shrunk in size in NPs + Laser group (Fig. 7b). The tumors tissues were isolated on day 18 (Fig. 7f), and the corresponding H&E and TUNEL immunofluorescent staining of tumor slices showed that tumor had been completely eliminated by synergistic phototherapy in NPs + Laser group (Additional file 1: Fig. S19). Whereas in other groups tumor volume was significantly increased (Fig. 7b). From the tumor temperature curve, we can see maximum temperature of the tumor in NPs + Laser is 53.5 ℃, which is higher than in Laser group (Fig. 7c and d). As for verifying the photodynamic effect of the A1 NPs in tumors, we performed ROS detection at the cell level and tissue level. Results showed ROS was produced in large quantities in tumor cells in NPs + Laser group (Additional file 1: Figs. S20b, S21). Mice weights were not significantly reduced after phototherapy (Fig. 7e). The above results confirmed that synergistic phototherapy based on A1 NPs had a superior performance to eliminate tumor.

Additionally, mature DC cells and CD8+ T cells were confirmed significantly higher in NPs + Laser group compared with the other control groups through quantitative analysis of CD8+ T cells in spleen and mature DC cells in tumor draining lymph nodes among four groups (Fig. 7g–j), which indicated that the synergistic phototherapy enabled by A1 NPs could induce antitumor T-cell immune responses. Next, the underlying mechanism of the anti-tumor immune responses of synergistic phototherapy was validated at cellular level. As reported, phototherapy often induces immunological cell death (ICD) of tumoral cells by releasing damage-associated molecular patterns (DAMPs) signals to trigger the maturation of DCs and activation of CD8+ T cells. To confirm our speculation, immunostimulatory molecules were examined after the phototherapy, including endoplasmic reticulum chaperone calreticulin (CRT), high-mobility group protein B1 (HMGB1) and heat shock protein (HSP) 70. As we can see in Additional file 1: Fig. S20, the cells treated with PBS nearly have no exposure of CRT, HMGB1 and HSP 70 (Additional file 1: Fig. S20c), while obvious green fluorescence was observed after incubated with A1 NPs and laser irradiation (Additional file 1: Fig. S20d), indicating A1 NPs can enhance the ICD induction effect. Exposure of these immune proteins was favorable for immune response triggered by phototherapy in vivo.

### A novel integrated strategy that achieves complete tumor eradication

**Fig. 8:**
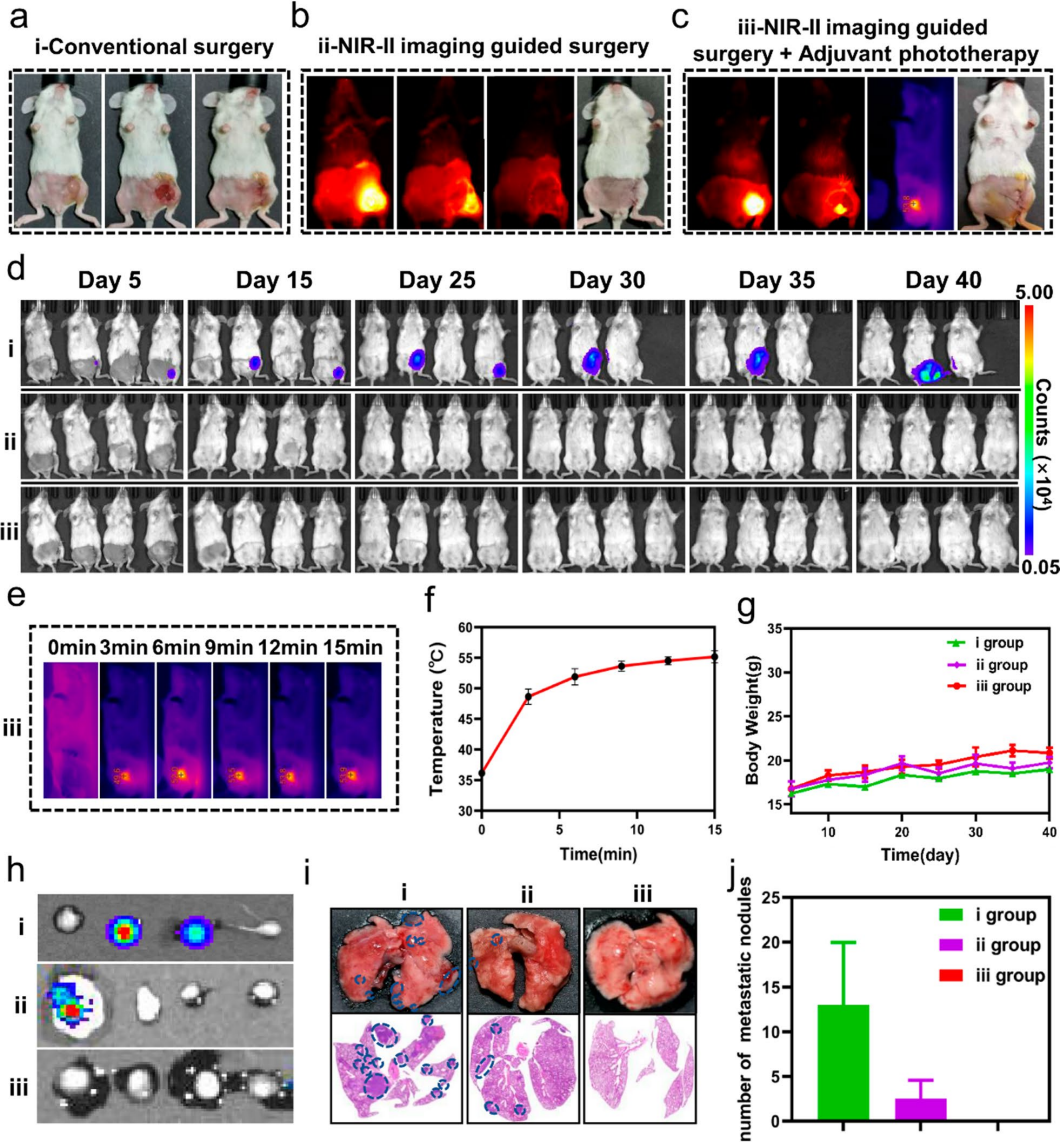
**a**–**c** Representative images of different surgery treatment (i–iii). **d** BLI images of mice in different surgery treatment group. **e**, **f** Photothermal images and heating curve of iii group mice at the residual tumor site. **g** The mice weight change in the different groups within 40 days. **h** BLI images of tumor-draining lymph nodes in the different groups. **i** Photograph and H&E staining of lung metastases in different groups. **j** Number of pulmonary metastatic nodules in different groups

We proposed a novel integrated strategy that NIR-II fluorescence-guided tumor resection and subsequent intraoperative fluorescence-guided phototherapy to eliminate residual microtumors based multifunctional A1 NPs. In this group (iii group), we firstly removed tumor under the guidance of NIR-II fluorescence imaging and the tiny tumor near the blood vessel remained. In order to avoid intraoperative bleeding, we used 808 nm lasers to irradiate the tumor cavity. We observed a rapid increase of the microtumors temperature in the tumor bed and up to 53.9° within 15 min (Fig. 8e and f). A marked increase in residual tumor temperature attributed to tumor-specific accumulation and excellent photothermal and photodynamic properties of A1 NPs. After the irradiation, the incisions were sutured (Fig. 8c). In conventional excisional surgery group (i group), the tumors were removed only by the same surgeon’ visual inspection and palpation. Until the surgeon thought the tumors have been completely removed, the incisions were sutured (Fig. 8a). In fluorescence imaging-guided surgery group (ii group), residual tumor lesions detected by NIR-II fluorescence imaging were surgically removed (Fig. 8b).

In conventional excisional surgery group, two mice developed local recurrence on day 5 and one of them was euthanized as tumor volumes had reached 2000 mm^3^ on day 30. Moreover, 13 lung metastasis nodules and 2 metastatic lymph nodes were detected. In fluorescence imaging-guided surgery group, no local recurrence was observed by BLI. But tumor metastasis signal was detected in one enlarged tumor draining lymph node and 2.5 lung metastasis nodules was confirmed by pathological examination. It was a pleasant surprise that no local recurrence or distant metastasis in any mice in iii group was observed (Fig. 8d, h–j). In addition, there was not significantly different in mice weight among three groups, proving the safety of the novel integrated strategy (Fig. 8g).

The above follow-up results showed the novel integrated strategy could significantly improve surgical outcomes. The reason can be attributed to the real-time guidance of NIR-II fluorescence imaging and enhanced anti-tumor immune effect induced by intraoperative adjuvant phototherapy.

### The biocompatibility and safety of A1 NPs

The systemic in vivo biocompatibility of the A1 NPs was evaluated in one week after various experiments. As shown in Additional file 1: Fig. S22a, there were no obvious damages in major tissues by histological analyses. In addition, blood biochemistry parameters of liver functions, renal functions and serum lipids showed no significant difference between different groups (Additional file 1: Fig. S22b). All results demonstrated the good biocompatibility of A1 NPs in vivo.

## Discussion

Incomplete tumor resection is the direct cause of the recurrence and metastasis after surgery. Aiming to improve surgical outcomes, we need to develop new intraoperative real-time imaging and treatment methods to accurately identify and eliminate residual tumor lesions. Motivated by the above needs, we tactfully designed and constructed a powerful “one-for-all” phototheranostic. More importantly, its imaging and therapeutic efficacy has been fully verified.

The current common practice for development of multifunctional phototheranostics is to integrate various components with individual functions into one platform [23]. Although this method is effective to a certain extent, it is unlike to achieve clinical applications because of complex preparation and formulation [24]. Aiming to realize clinical applications, we plan to construct a simple and one-for-all phototheranostics. However, this is still a challenging task because some of these processes are seriously competitive to each other from the perspective of energy consumption. According to the Jablonski diagram, upon photoexcitation, an energetically excited state is formed, which is then consumed through both radiative decay (emission of photon for FLI) or non-radiative decay (for PTT and PDT) [25, 26]. How to tactfully regulate the balance between radiative and nonradiative energy dissipations is the key for development of multifunctional phototheranostics. Aggregation-induced emission (AIE)-active luminogens (AIEgens) have been developed for this attempt by tactfully controlling molecular motions for balancing radiative and nonradiative decays. While these fluorophores have good fluorescence quantum yield and excellent photothermal conversion efficiency, it cannot guarantee good performance in imaging and therapy. This is because the total dissipation energy is also highly dependent on the light absorption of the molecule. Unfortunately, most AIEgens have an inferior absorption coefficient, because molecular distortion inevitably destroys the conjugation [27]. In this study, we designed and synthesized a D1–π–A–D2–R type fluorophore A1 though integration of a planar π-conjugated units and twisted motifs into one molecule, endowing A1 with a significantly enhanced ε (4.761 * 10^− 4^ M^− 1^ cm^− 1^). The ε of A1 exceeds that of most reported AIE molecules [28]. In addition, in this experiment, we compared A1 with two representative D–A–D structure molecules A2 and A3. The results shows that the A1 molecule has a higher molar absorption coefficient, which highlights the asymmetric structure may be a good design strategy for developing multifunctional fluorophores. In all, simultaneously shows high absolute quantum yield (1.23%), excellent photothermal conversion efficiency (55.3%), high molar absorption coefficient and moderate singlet oxygen generation performance. These superior performances lay a foundation for their future medical applications.

We successfully achieved detection of the residual small tumors in tumor bed by intravenous administration of A1 NPs in orthotopic breast mice models. The average SBR of residual tumor lesion was 2.1 and the smallest detectable residual tumor lesion was 2 mm that can’t be detected by eye examination. Using the fluorescence imaging, the surgeon may alter the initial surgical plan and perform additional resection during surgery, avoiding local tumor recurrence after surgery. We all know that the precise identification of metastatic lymph nodes intraoperative is a key clinical problem. If the metastatic lymph nodes can be accurately identified during surgery, extensive lymph node dissection can be avoided which can cause lymphedema. Most notable in our study was the finding that metastatic lymph nodes could be detected specifically through intravenous injection of the A1 NPs. As for the relevant mechanisms for preferential uptake of A1 NPs in the metastatic lymph nodes is not very clear. The most likely hypothesis involves the enhanced permeability and retention (EPR) effect due to neoangiogenesis in metastatic lymph nodes. The mechanisms need to be further explored.

In many clinical practices, complete tumor resection is difficult such as ovarian carcinoma, neoplasms of central nervous system and lymphoma. For these situations, we proposed a novel integrated strategy that NIR-II fluorescence-guided primary tumor resection and adjuvant intraoperative phototherapy elimination of unresectable residual tumors under fluorescence-guided. We observed this new strategy achieved a good tumor treatment outcome with none of the cases suffered recurrence or metastasis during postoperative follow-up. The potential reasons lie in: on the one hand, NIR-II fluorescence imaging clearly image tumor boundary and guide tumor resection. Even if there were some unresectable residual tumors that have not been removed during the operation, they could be detected and eliminated by targeted phototherapy under the guidance of fluorescence; on the other hand, phototherapy has been proved to enhance systemic antitumor T cell immunity by inducing ICD of cancer cells to eradicate disseminated tumor cells, and ultimately inhibit local recurrence and distant metastasis [20, 29]. Our preliminary results demonstrated the feasibility and effectiveness of this strategy. This new strategy is simple to carry out only though a single intravenous injection of the A1 NPs. If combined with immune therapeutics such as PD-1/PD-L1 inhibitors and CTLA4 antibody [30, 31], this strategy may achieve even better results. In a recent study, researchers induced the stronger immune responses by using PDT and PTT in sequence without additional therapeutics [32]. A large number of studies have proved the ability of phototherapy to destroy tumor cells, but limited by its penetration depth, it has not been used in clinic. In the current study, we used phototherapy in elimination of intraoperative residual tumors, which circumvents its limitations and facilitates its clinical translation. At present, there have been relevant research reports on postoperative phototherapy to eliminate residual tumors, but there is no report on an integrated strategy that can guide tumor resection during surgery and eliminate residual tumors after surgery. We believe that this new strategy can optimize debulking surgery and achieve better tumor cell control.

It should be noted that the tumor accumulation of A1 NPs was mainly through the EPR effect. We next plan to link A1 NPs with some tumor targeting antibodies, such as anti-EGFR, anti-PMSA, anti-VEGF antibodies to construct tumor-targeted theranostic agents, as combination of both passive and active targeting approaches might further improve theranostic effect. Furthermore, the study was only performed in orthotopic 4T1 mice model, and additional tumor models are needed to fully verify the A1 NPs imaging and treatment effects. In addition, we also will try other modal imaging to guide surgical resection, such as Cerenkov luminescence imaging [33–36].

At present, a series of nanotechnology has entered clinical trials and began to play a role in tumor diagnosis and treatment [37–39]. The biocompatibility of AIE probes has been verified in Marmosets [40]. Additionally, our preliminary data suggest A1 NPs is safe at both cell and live animal level. The nanoprobe developed has the potential to be translated in clinic to improve surgical results.

## Conclusions

In summary, we successfully develop a powerful D1–π–A–D2–R type phototheranostic based on aggregation-induced emission (AIE) allowing all of NIR-II FLI, PTT, and PDT capabilities. The efficacy of each function is well balanced and maximized (NIR-II quantum yield: 1.23%; photothermal conversion efficiency: 55.3%; high reactive oxygen species generation). By constructing orthotopic cancer model in mice, tumor and metastatic lymph nodes can be completely resected under NIR-II fluorescence-imaging guided using the A1 NPs. Furthermore, when there are unresectable residual tumor lesions during the operation, a novel integrated strategy can be performed to improve surgical outcomes.

## Supplementary Information

**Additional file 1.Scheme S1. **The synthetic route toward A1.** Fig S1.** ^1^H NMR spectroscopy of compound 1. **Fig S2.** ^13^C NMR spectroscopy of compound 1. **Fig S3.** ^1^H NMR spectroscopy of compound 2. **Fig S4.** ^13^C NMR spectroscopy of compound 2. **Fig S5.** MALDI-TOF-MS measurement of compound 2. **Fig S6.** ^1^H NMR spectroscopy of compound 3. **Fig S7.** MS spectroscopy of compound 3. **Fig S8.** ^1^H NMR spectroscopy of compound 5. **Fig S9.** MALDI-TOF-MS measurement of compound 5. **Fig S10.** ^1^H NMR spectroscopy of compound A1. **Fig S11.** MALDI-TOF-MS measurement of compound A1. **Fig S12. a**) The hydrodynamic particle diameter and **b**) the fluorescence intensity of A1 NPs in PBS during 14days (0h, 1d, 3d, 7d and 14d). Fig S13. NIR-II fluorescence images of 4T1 tumor-bearing mice at various time points after tail-vein administration of the A1 NPs. **Fig S14.** NIR-II fluorescence images of tumor frozen sections. **Fig S15. a, d**) NIR-II images of orthotopic 4T1 breast tumor resection procedure. **b, c, e, f**) H&E staining images of excised tumor and residual lesion. **Fig S16.** BLI images detected two positive sciatic lymph nodes in orthotopic breast cancer mouse. A clear fluorescent signal matched well with that of the bioluminescence imaging (BLI). Positive lymph nodes were resected under the NIR-II fluorescence imaging. Metastatic lymph nodes were confirmed by H&E staining. **Fig S17.** H&E staining of remaining enlarged lymph nodes in sciatic lymph node metastasis mice. **Fig S18.** H&E staining of remaining enlarged lymph nodes in axillary lymph node metastasis mice. **Fig S19.** Representative H&E staining and CLSM images of tumors after TUNEL staining. **Fig S20. a**) Cytotoxicity assays of different concentrations A1 NPs in the dark (incubation for 24 h) or under laser exposure for 5 min (808 nm, 1 W/cm^2^). **b**) ROS generation detection from 4T1-luc cells with different treatment using DCFH-DA. **c**) Immunofluorescence staining of CRT, HMGB1 and HSP 70 after 4T1-luc cells treated with PBS. **d**) Immunofluorescence staining of CRT, HMGB1 and HSP 70 after 4T1-luc cells treated with A1+Laser. **Fig S21.** (**a**) Representative merge images include DAPI staining (blue) and ROS staining (red) of tumor tissue after the different treatments; (**b**) Fluorescence intensity analysis of ROS in different groups. (**P < 0.01, ***P <0.001). **Fig S22. a**) H&E staining of the main organs from the mice in different groups. **b**) Blood biochemistry analysis of the mice receiving different treatments.

## Data Availability

All data generated or analysed during this study are included in this published article and its additional file.

## References

[1] Arbyn M, Redman CWE, Verdoodt F, Kyrgiou M, Tzafetas M, Ghaem-Maghami S, Petry K-U, Leeson S, Bergeron C, Nieminen P (2017). Incomplete excision of cervical precancer as a predictor of treatment failure: a systematic review and meta-analysis. Lancet Oncol.

[2] van der Werf LR, Cords C, Arntz I, Belt EJT, Cherepanin IM, Coene PLO, van der Harst E, Heisterkamp J, Langenhoff BS, Lamme B (2019). Population-based study on risk factors for tumor-positive resection margins in patients with gastric cancer. Ann Surg Oncol.

[3] Moran MS, Schnitt SJ, Giuliano AE, Harris JR, Khan SA, Horton J, Klimberg S, Chavez-MacGregor M, Freedman G, Houssami N (2014). Society of Surgical Oncology-American Society for Radiation Oncology consensus guideline on margins for breast-conserving surgery with whole-breast irradiation in stages I and II invasive breast cancer. Int J Radiat Oncol Biol Phys.

[4] Cutress RI, McIntosh SA, Potter S, Goyal A, Kirwan CC, Harvey J, Francis A, Carmichael AR, Vidya R, Vaidya JS (2018). Opportunities and priorities for breast surgical research. Lancet Oncol.

[5] Boogerd LSF, Hoogstins CES, Schaap DP, Kusters M, Handgraaf HJM, van der Valk MJM, Hilling DE, Holman FA, Peeters K, Mieog JSD (2018). Safety and effectiveness of SGM-101, a fluorescent antibody targeting carcinoembryonic antigen, for intraoperative detection of colorectal cancer: a dose-escalation pilot study. Lancet Gastroenterol Hepatol.

[6] van Keulen S, Nishio N, Fakurnejad S, Birkeland A, Martin BA, Lu G, Zhou Q, Chirita SU, Forouzanfar T, Colevas AD (2019). The clinical application of fluorescence-guided surgery in head and neck cancer. J Nucl Med.

[7] Lauwerends LJ, van Driel P, Baatenburg de Jong RJ, Hardillo JAU, Koljenovic S, Puppels G, Mezzanotte L, Lowik C, Rosenthal EL, Vahrmeijer AL, Keereweer S (2021). Real-time fluorescence imaging in intraoperative decision making for cancer surgery. Lancet Oncol.

[8] Vahrmeijer AL, Hutteman M, van der Vorst JR, van de Velde CJ, Frangioni JV (2013). Image-guided cancer surgery using near-infrared fluorescence. Nat Rev Clin Oncol.

[9] Tichauer KM, Samkoe KS, Gunn JR, Kanick SC, Hoopes PJ, Barth RJ, Kaufman PA, Hasan T, Pogue BW (2014). Microscopic lymph node tumor burden quantified by macroscopic dual-tracer molecular imaging. Nat Med.

[10] Kennedy GT, Azari FS, Bernstein E, Marfatia I, Din A, Kucharczuk JC, Low PS, Singhal S (2021). Targeted intraoperative molecular imaging for localizing nonpalpable tumors and quantifying resection margin distances. JAMA Surg.

[11] Lamberts LE, Koch M, de Jong JS, Adams ALL, Glatz J, Kranendonk MEG, van TerwisschaScheltinga AGT, Jansen L, de Vries J, Lub-de Hooge MN (2017). Tumor-specific uptake of fluorescent bevacizumab-IRDye800CW microdosing in patients with primary breast cancer: a phase I feasibility study. Clin Cancer Res.

[12] Whitley MJ, Cardona DM, Lazarides AL, Spasojevic I, Ferrer JM, Lee CL, Snuderl M, Blazer DG, Hwang ES (2016). A mouse-human phase 1 co-clinical trial of a protease-activated fluorescent probe for imaging cancer. Sci Transl Med.

[13] He S, Song J, Qu J, Cheng Z (2018). Crucial breakthrough of second near-infrared biological window fluorophores: design and synthesis toward multimodal imaging and theranostics. Chem Soc Rev.

[14] Antaris AL, Chen H, Cheng K, Sun Y, Hong G, Qu C, Diao S, Deng Z, Hu X, Zhang B (2016). A small-molecule dye for NIR-II imaging. Nat Mater.

[15] Xu P, Hu L, Yu C, Yang W, Kang F, Zhang M, Jiang P, Wang J (2021). Unsymmetrical cyanine dye via in vivo hitchhiking endogenous albumin affords high-performance NIR-II/photoacoustic imaging and photothermal therapy. J Nanobiotechnol.

[16] Hu Z, Fang C, Li B, Zhang Z, Cao C, Cai M, Su S, Sun X, Shi X, Li C (2020). First-in-human liver-tumour surgery guided by multispectral fluorescence imaging in the visible and near-infrared-I/II windows. Nat Biomed Eng.

[17] Tsonis O, Gkrozou F, Vlachos K, Paschopoulos M, Mitsis MC, Zakynthinakis-Kyriakou N, Boussios S, Pappas-Gogos G (2020). Upfront debulking surgery for high-grade serous ovarian carcinoma: current evidence. Ann Transl Med.

[18] Orecchia R, Veronesi U, Maisonneuve P, Galimberti VE, Lazzari R, Veronesi P, Jereczek-Fossa BA, Cattani F, Sangalli C, Luini A (2021). Intraoperative irradiation for early breast cancer (ELIOT): long-term recurrence and survival outcomes from a single-centre, randomised, phase 3 equivalence trial. Lancet Oncol.

[19] Chen Q, Xu L, Liang C, Wang C, Peng R, Liu Z (2016). Photothermal therapy with immune-adjuvant nanoparticles together with checkpoint blockade for effective cancer immunotherapy. Nat Commun.

[20] Zeng Z, Zhang C, Li J, Cui D, Jiang Y, Pu K (2021). Activatable polymer nanoenzymes for photodynamic immunometabolic cancer therapy. Adv Mater.

[21] Wang S, Ma X, Hong X, Cheng Y, Tian Y, Zhao S, Liu W, Tang Y, Zhao R, Song L (2018). Adjuvant photothermal therapy inhibits local recurrences after breast-conserving surgery with little skin damage. ACS Nano.

[22] Wei Q, Arami H, Santos HA, Zhang H, Li Y, He J, Zhong D, Ling D, Zhou M (2021). Intraoperative assessment and photothermal ablation of the tumor margins using gold nanoparticles. Adv Sci (Weinh).

[23] Vankayala R, Hwang KC (2018). Near-infrared-light-activatable nanomaterial-mediated phototheranostic nanomedicines: an emerging paradigm for cancer treatment. Adv Mater.

[24] Chen C, Ou H, Liu R, Ding D (2020). Regulating the photophysical property of organic/polymer optical agents for promoted cancer phototheranostics. Adv Mater.

[25] Ni X, Zhang X, Duan X, Zheng HL, Xue XS, Ding D (2019). Near-infrared afterglow luminescent aggregation-induced emission dots with ultrahigh tumor-to-liver signal ratio for promoted image-guided cancer surgery. Nano Lett.

[26] Qi J, Chen C, Zhang X, Hu X, Ji S, Kwok RTK, Lam JWY, Ding D, Tang BZ (2018). Light-driven transformable optical agent with adaptive functions for boosting cancer surgery outcomes. Nat Commun.

[27] Qi J, Duan X, Liu W, Li Y, Cai Y, Lam JWY, Kwok RTK, Ding D, Tang BZ (2020). Dragonfly-shaped near-infrared AIEgen with optimal fluorescence brightness for precise image-guided cancer surgery. Biomaterials.

[28] Liu S, Li Y, Kwok RTK, Lam JWY, Tang BZ (2020). Structural and process controls of AIEgens for NIRII theranostics. Chem Sci.

[29] Yan S, Zeng X, Tang Y, Liu BF, Wang Y, Liu X (2019). Activating antitumor immunity and antimetastatic effect through polydopamine-encapsulated core-shell upconversion nanoparticles. Adv Mater.

[30] Min Y, Roche KC, Tian S, Eblan MJ, McKinnon KP, Caster JM, Chai S, Herring LE, Zhang L, Zhang T (2017). Antigen-capturing nanoparticles improve the abscopal effect and cancer immunotherapy. Nat Nanotechnol.

[31] Zhu G, Mei L, Vishwasrao HD, Jacobson O, Wang Z, Liu Y, Yung BC, Fu X, Jin A, Niu G (2017). Intertwining DNA-RNA nanocapsules loaded with tumor neoantigens as synergistic nanovaccines for cancer immunotherapy. Nat Commun.

[32] Yang J, Hou M, Sun W, Wu Q, Xu J, Xiong L, Chai Y, Liu Y, Yu M, Wang H (2020). Sequential PDT and PTT Using dual-modal single-walled carbon nanohorns synergistically promote systemic immune responses against tumor metastasis and relapse. Adv Sci (Weinh).

[33] Hu Z, Chen X, Liang J, Qu X, Chen D, Yang W, Wang J, Cao F, Tian J (2012). Single photon emission computed tomography-guided Cerenkov luminescence tomography. J Appl Phys.

[34] Liu M, Zheng S, Zhang X, Guo H, Shi X, Kang X, Qu Y, Hu Z, Tian J (2018). Cerenkov luminescence imaging on evaluation of early response to chemotherapy of drug-resistant gastric cancer. Nanomedicine.

[35] Qin C, Zhong J, Hu Z, Yang X, Tian J (2012). Recent advances in cerenkov luminescence and tomography imaging. IEEE J Sel Top Quantum Electron.

[36] Yang W, Qin W, Hu Z, Suo Y, Zhao R, Ma X, Ma W, Wang T, Liang J, Tian J, Wang J (2012). Comparison of Cerenkov luminescence imaging (CLI) and gamma camera imaging for visualization of let-7 expression in lung adenocarcinoma A549 Cells. Nucl Med Biol.

[37] de Valk KS, Deken MM, Handgraaf HJM, Bhairosingh SS, Bijlstra OD, van Esdonk MJ, van TerwisschaScheltinga AGT, Valentijn A, March TL, Vuijk J (2020). First-in-human assessment of cRGD-ZW800-1, a Zwitterionic, Integrin-targeted, near-infrared fluorescent peptide in colon carcinoma. Clin Cancer Res.

[38] Basa-Denes O, Angi R, Karpati B, Jordan T, Otvos Z, Erdosi N, Ujhelyi A, Ordasi B, Molnar L, McDermott J (2019). Dose escalation study to assess the pharmacokinetic parameters of a nano-amorphous oral sirolimus formulation in healthy volunteers. Eur J Drug Metab Pharmacokinet.

[39] Voskuil FJ, Steinkamp PJ, Zhao T, Vegt Bvd, Koller M, Doff JJ, Jayalakshmi Y, Hartung JP, Gao J, Sumer BD (2020). Exploiting metabolic acidosis in solid cancers using a tumor-agnostic pH-activatable nanoprobe for fluorescence-guided surgery. Nat Commun.

[40] Feng Z, Bai S, Qi J, Sun C, Zhang Y, Yu X, Ni H, Wu D, Fan X, Xue D (2021). Biologically excretable aggregation-induced emission dots for visualizing through the marmosets intravitally: horizons in future clinical nanomedicine. Adv Mater.

