# Supplementary material for: Novel multifunctional NIR-II aggregation-induced emission nanoparticles-assisted intraoperative identification and elimination of residual tumor
Source: J Nanobiotechnology. 2022 Mar 19;20:143. doi: 10.1186/s12951-022-01325-9 (PMC8934469; doi:10.1186/s12951-022-01325-9)
Supplement: Supplementary file 1 — Additional file 1.Scheme S1. The synthetic route toward A1. Fig S1. 1H NMR spectroscopy of compound 1. Fig S2. 13C NMR spectroscopy of compound 1. Fig S3. 1H NMR spectroscopy of compound 2. Fig S4. 13C NMR spectroscopy of compound 2. Fig S5. MALDI-TOF-MS measurement of compound 2. Fig S6. 1H NMR spectroscopy of compound 3. Fig S7. MS spectroscopy of compound 3. Fig S8. 1H NMR spectroscopy of compound 5. Fig S9. MALDI-TOF-MS measurement of compound 5. Fig S10. 1H NMR spectroscopy of compound A1. Fig S11. MALDI-TOF-MS measurement of compound A1. Fig S12. a) The hydrodynamic particle diameter and b) the fluorescence intensity of A1 NPs in PBS during 14days (0h, 1d, 3d, 7d and 14d). Fig S13. NIR-II fluorescence images of 4T1 tumor-bearing mice at various time points after tail-vein administration of the A1 NPs. Fig S14. NIR-II fluorescence images of tumor frozen sections. Fig S15. a, d) NIR-II images of orthotopic 4T1 breast tumor resection procedure. b, c, e, f) H&E staining images of excised tumor and residual lesion. Fig S16. BLI images detected two positive sciatic lymph nodes in orthotopic breast cancer mouse. A clear fluorescent signal matched well with that of the bioluminescence imaging (BLI). Positive lymph nodes were resected under the NIR-II fluorescence imaging. Metastatic lymph nodes were confirmed by H&E staining. Fig S17. H&E staining of remaining enlarged lymph nodes in sciatic lymph node metastasis mice. Fig S18. H&E staining of remaining enlarged lymph nodes in axillary lymph node metastasis mice. Fig S19. Representative H&E staining and CLSM images of tumors after TUNEL staining. Fig S20. a) Cytotoxicity assays of different concentrations A1 NPs in the dark (incubation for 24 h) or under laser exposure for 5 min (808 nm, 1 W/cm2). b) ROS generation detection from 4T1-luc cells with different treatment using DCFH-DA. c) Immunofluorescence staining of CRT, HMGB1 and HSP 70 after 4T1-luc cells treated with PBS. d) Immunofluorescence staining of CRT, HMGB [file 12951_2022_1325_MOESM1_ESM.docx]

Additional Information

**Novel Multifunctional NIR-II Aggregation-Induced Emission Nanoparticles-Assisted Intraoperative Identification and Elimination of** **Residual Tumor**

Qiaojun Qu^1, 2^, Zeyu Zhang^2, 3^, Xiaoyong Guo^2, 4, 5^, Junying Yang^6^, Caiguang Cao^2, 7^, Changjian Li^3^, Pengfei Xu^8, 9, *^, Zhenhua Hu^2, 7, *^, Jie Tian^1, 2, 3, 7, *^

^1^College of Medical Imaging, Shanxi Medical University, Taiyuan 030001, China

^2^CAS Key Laboratory of Molecular Imaging, Beijing Key Laboratory of Molecular Imaging, The State Key Laboratory of Management and Control for Complex Systems, Institute of Automation, Chinese Academy of Sciences, Beijing 100190, China

^3^Beijing Advanced Innovation Center for Big Data-Based Precision Medicine, School of Medicine and Engineering, Beihang University, Beijing 100191, China

^4^Department of Gastroenterology, the Third Medical Centre, Chinese PLA General Hospital, Beijing100190, China.

^5^Anhui Medical University, Hefei 230000, China.

^6^Department of Hepatobiliary Surgery, Zhujiang Hospital, Southern Medical University, Guangzhou 510280, China

^7^School of Artificial Intelligence, University of Chinese Academy of Sciences, Beijing 100049, China

^8^Departments of Diagnostic Radiology, Surgery, Chemical and Biomolecular Engineering, and Biomedical Engineering, Yong Loo Lin School of Medicine and Faculty of Engineering, National University of Singapore, Singapore, 119074, Singapore

^9^Institute of Clinical Pharmacy & Pharmacology, Jining First People’s Hospital, Jining Medical University, Jining 272000, P.R. China

*Corresponding authors.

E-mail addresses: [pengfeixuscu@outlook.com](mailto:pengfeixuscu@outlook.com); zhenhua.hu@ia.ac.cn; jie.tian@ia.ac.cn.

**Experimental Section**

**Synthesis A1** **and Characterization of compound A1**

Synthesis and Characterization of compound **2**. To a solution of bis(triphenylphosphine)palladium (II) dichloride (13 mg, 0.0175 mmol), and 1,1,1,2,2,2-hexabutyldistannane (638 mg, 1.1 mmol) in dioxane (20 mL) was added compound **1** (380 mg, 1.0 mmol) under an inert atmosphere (N_2_). The reaction mixture was heated in an oil bath at 80 ^o^C for 12 hours. When the reaction was finished, the crude product was purified by basic alumina chromatography (petroleum ether: dichloromethane = 10:1 v/v). The product was obtained as yellow oil (407mg, 69 %). Then, a mixture of 4,7-dibromobenzo[1,2-c:4,5-c']bis([1,2,5]thiadiazole) (260 mg, 0.75 mmol), all the obtained stannylation product, Pd(PPh_3_)_4_ (12 mg, 0.1 mmol) and anhydrous toluene (20 mL) was stirred at 100^o^C under argon atmosphere overnight. After the reaction was completed, the solvent was removed by rotary evaporator. The crude product was purified by silica column with petroleum ether /dichloromethane (v: v = 1:2) as the eluent to afford compound **2** as green powders (172 mg, 44% yield).^1^H NMR (400 MHz, Chloroform-*d*) δ 9.95 (s, 2H), 8.35 – 8.22 (m, 2H), 7.90 – 7.78 (m, 4H), 7.40 (d, *J* = 8.7 Hz, 2H), 7.35 (d, *J* = 8.6 Hz, 4H). ^13^C NMR (101 MHz, CDCl_3_) δ 190.52, 153.76, 151.85, 151.70, 146.54, 133.57, 132.02, 131.47, 131.39, 125.56, 123.87. MALDI-TOF [Calcd. for C_26_H_14_BrN_5_O_2_S_2_^+^: 570.98, found: m/z 571.895.]

Synthesis and Characterization of compound **5**. Compound **3** (380 mg, 1.0 mmol) was dissolved in dioxane (20 mL) under an inert atmosphere (N_2_). Then, bis(triphenylphosphine)palladium (II) dichloride (13 mg, 0.0175 mmol) and 1,1,1,2,2,2-hexabutyldistannane (638 mg, 1.1 mmol) were added. The reaction mixture was heated in an oil bath at 80 ^o^C for 12 hours. When the reaction was finished, the crude product was purified by basic alumina chromatography (petroleum ether: dichloromethane = 5:1 v/v). The product was obtained as yellow oil (218mg, 41%). Then, a mixture of 4,7-dibromobenzo[1,2-c:4,5-c']bis([1,2,5]thiadiazole) (115mg, 0.2 mmol), all the obtained stannylation product, Pd(PPh_3_)_4_ (12 mg, 0. 1 mmol) and anhydrous toluene (20 mL) was stirred at 100 ^o^C under argon atmosphere overnight. After the reaction was completed, the solvent was removed by rotary evaporator. The crude product was purified by silica column with dichloromethane/methanol (v: v = 50:1) as the eluent to afford compound **5** as green powders (63 mg, 43% yield).^1^H NMR (400 MHz, Chloroform-*d*) δ 9.94 (s, 2H), 8.90 (s, 2H), 8.35 (d, *J* = 8.0 Hz, 2H), 8.04 (s, 1H), 7.84 (d, *J* = 8.3 Hz, 4H), 7.53 (d, *J* = 8.5 Hz, 1H), 7.37 (dd, *J* = 16.8, 8.0 Hz, 6H), 7.13 (s, 1H), 6.64 (d, *J* = 8.7 Hz, 1H), 3.55 – 3.39 (m, 5H), 1.33 (s, 6H). MALDI-TOF [Calcd. for C_40_H_30_N_6_O_4_S_2_^-^: 733.18, found: m/z 733.625.]

Synthesis and Characterization of compound **A1**. To a 50 mL round bottomed flask was added with compound **5** (73 mg, 0.1 mmol), compound **6** (200 mg, 0.3 mmol), THF (25mL), and the resulting solution was chilled to 0-5 ^o^C in an ice bath. Potassium tert-butoxide (134 mg, 1.2 mmol) was added and the reaction mixture was stirred at 0-5 ^o^C for 1 hour and then warmed to ambient temperature. The reaction was monitored by TLC analysis and it was completed in 12 hours. The reaction mixture was concentrated in vacuo and the residue was purified by silica gel chromatography (petroleum ether: dichloromethane = 8:1 v/v) to give a bright yellow solid 1 (70 mg, 51% yield). ^1^H NMR (600 MHz, Chloroform-*d*) δ 8.73 (d, *J* = 37.9 Hz, 2H), 8.17 (s, 2H), 7.92 (d, *J* = 32.3 Hz, 1H), 7.42 – 6.96 (m, 49H), 6.58 – 6.39 (m, 4H), 3.37 (s, 4H), 1.48 (s, 7H). MALDI-TOF [Calcd. for C_96_H_70_N_6_O_4_S_2_^+^: 1390.50, found: m/z 1391.541.] To evaluate the AIE properties of A1, we prepared eight organic solutions of A1 with different water fraction (f_w_) and recorded their PL spectra respectively. Beer’s law is an optical fundamental law and is expressed as: ε= A/dc. Light absorbance (A) is proportional to absorption coefficient (ε), optical path length (d), and concentration (c). The optical path length d of a sample solution is expressed in centimeters, and c is expressed in molar concentration. Absorption coefficient is multiplied by the reciprocal of centimeter to the reciprocal of molar concentration M^−1^ cm^−1^as a unit. The characterization results of key compounds are shown in Fig S1-Fig S11.

Scheme S1. The synthetic route toward A1.


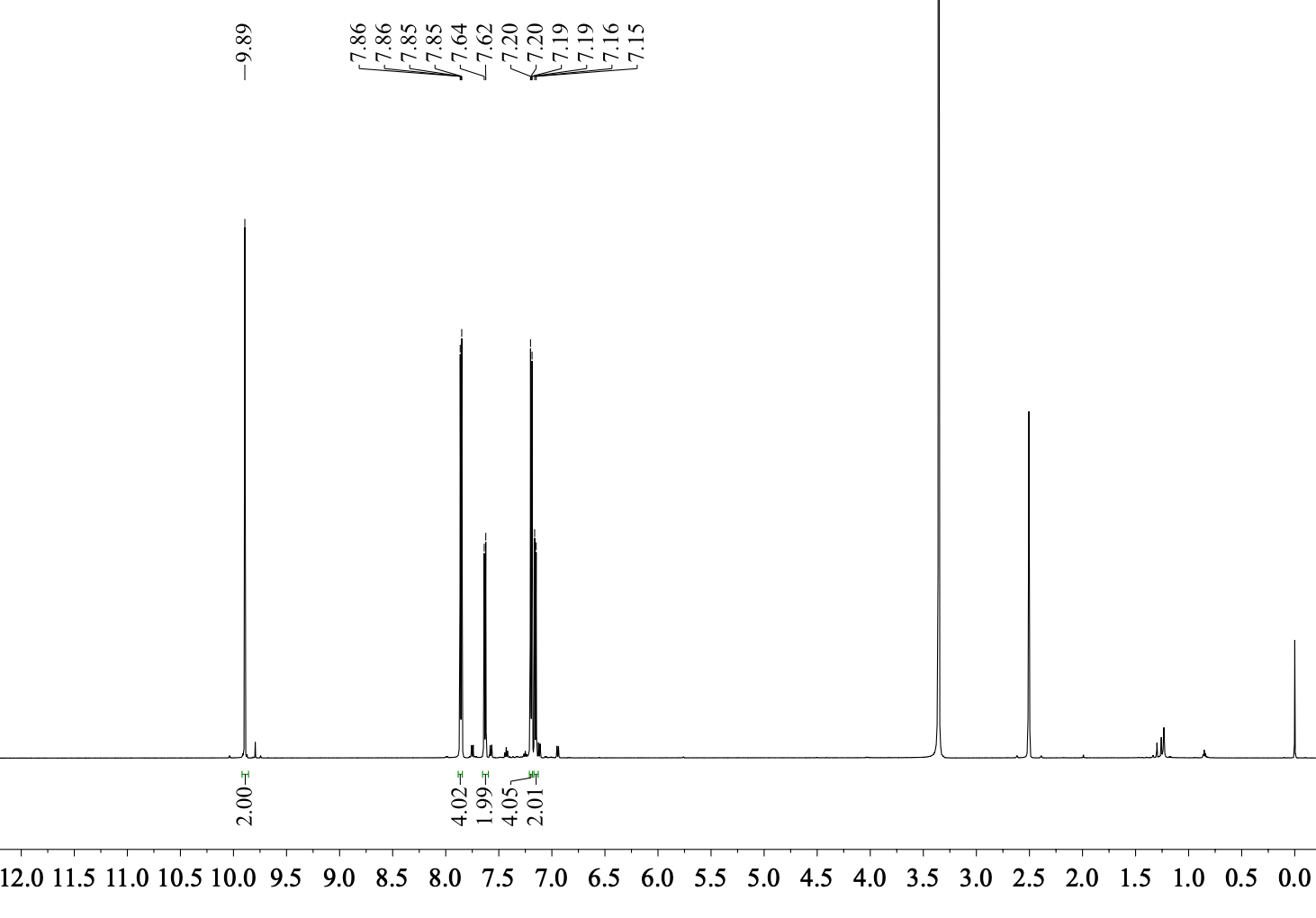


Fig S1. ^1^H NMR spectroscopy of compound **1**.


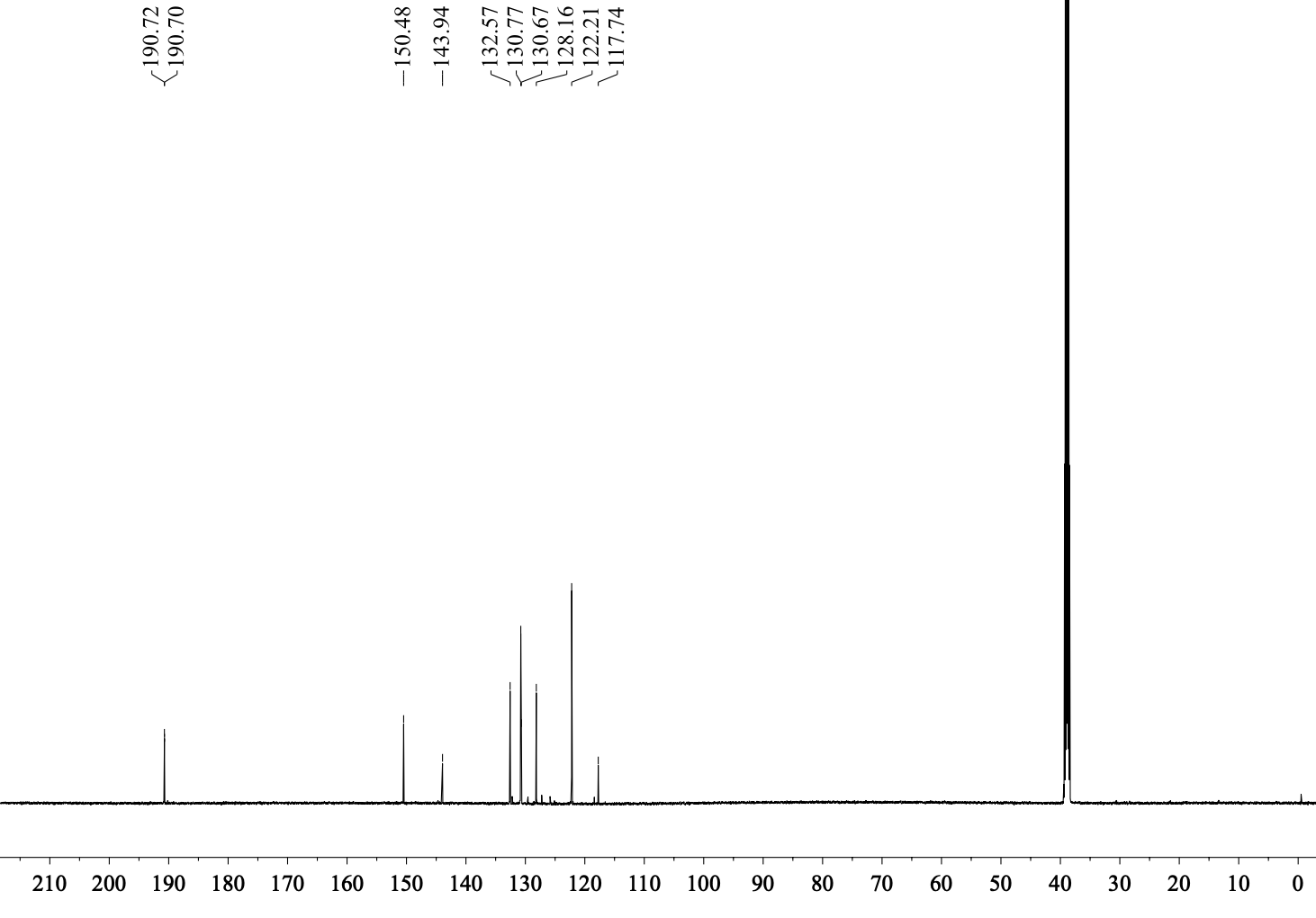


Fig S2. ^13^C NMR spectroscopy of compound **1**.


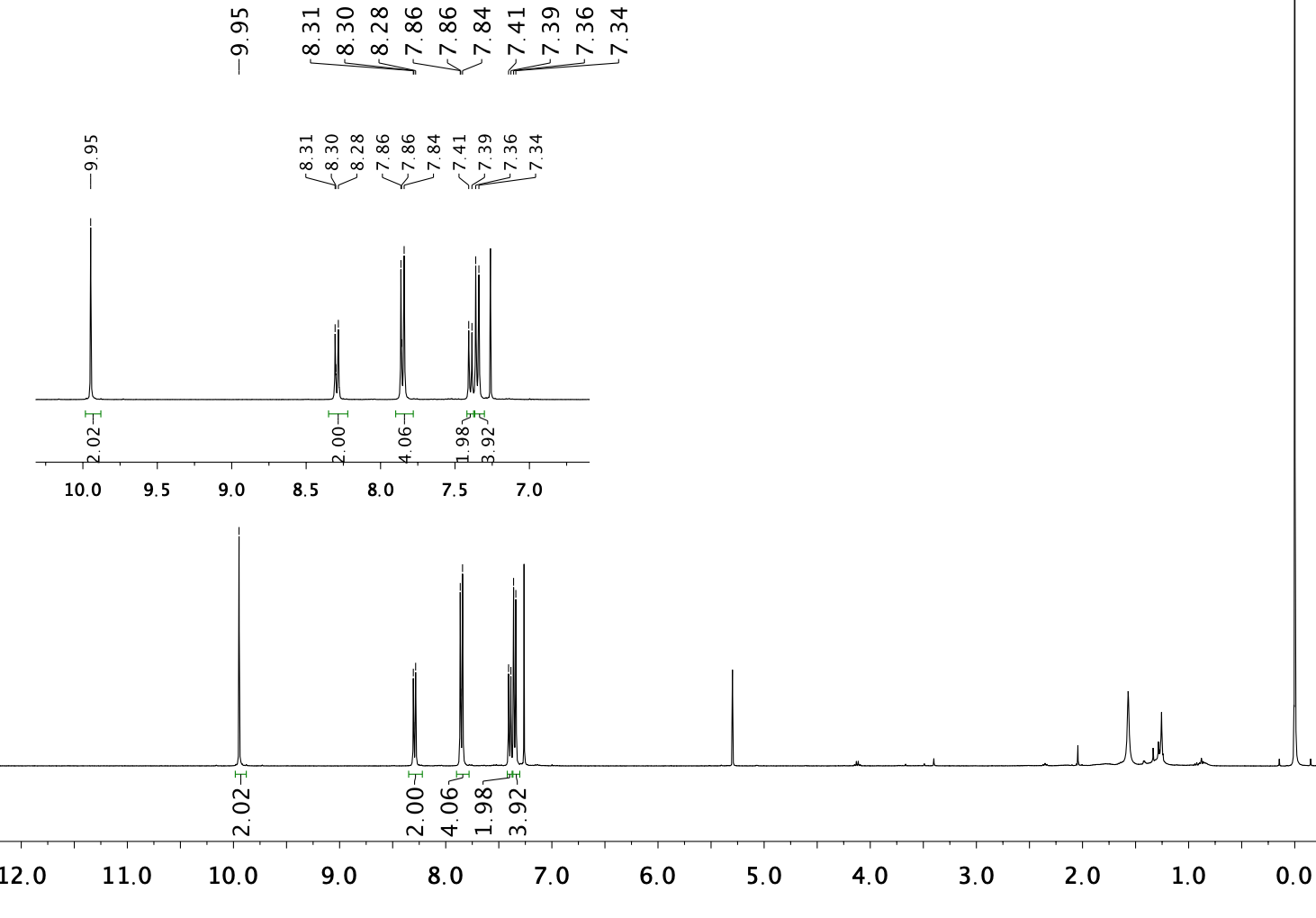


Fig S3. ^1^H NMR spectroscopy of compound **2**.


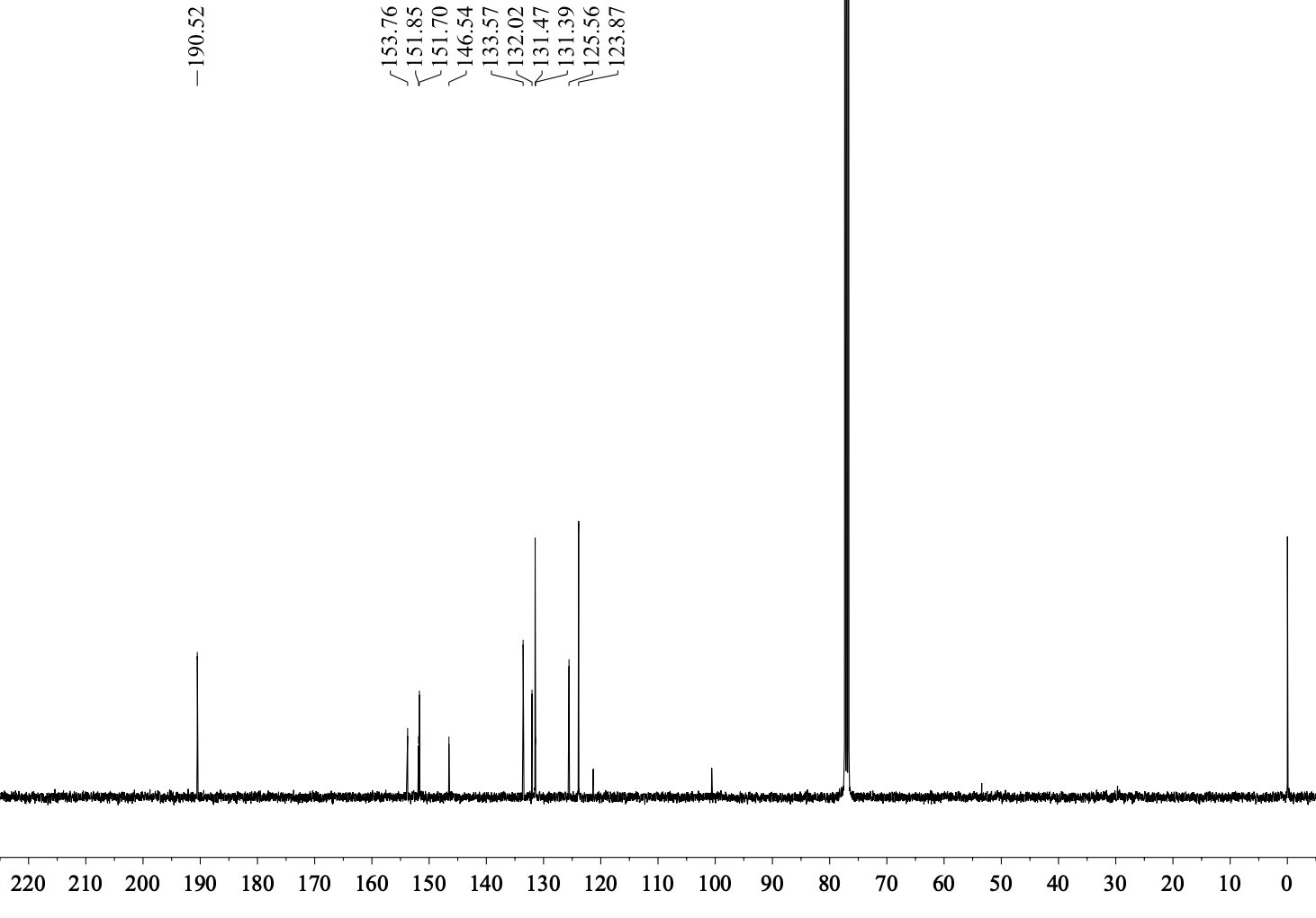


Fig S4. ^13^C NMR spectroscopy of compound **2**.


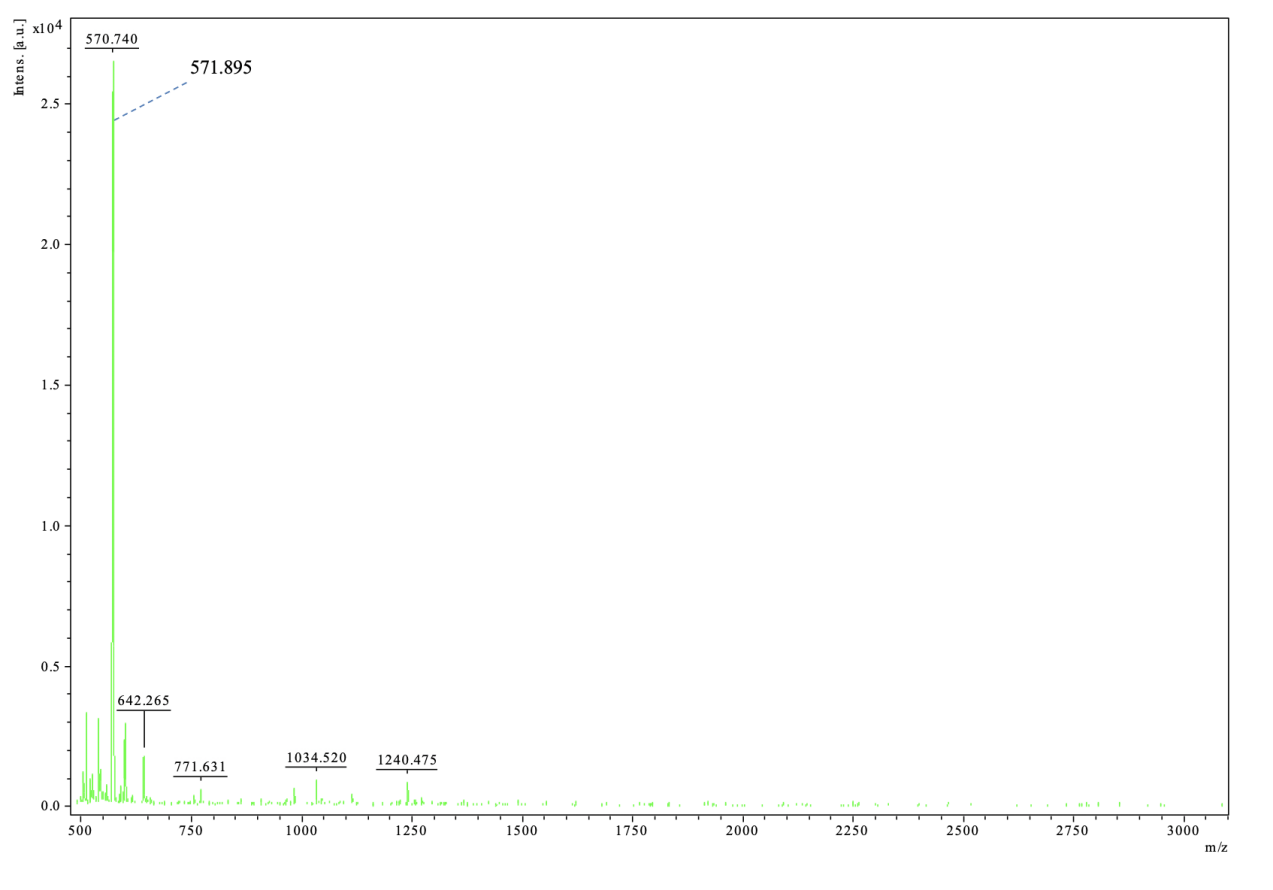


Fig S5. MALDI-TOF-MS measurement of compound **2**.


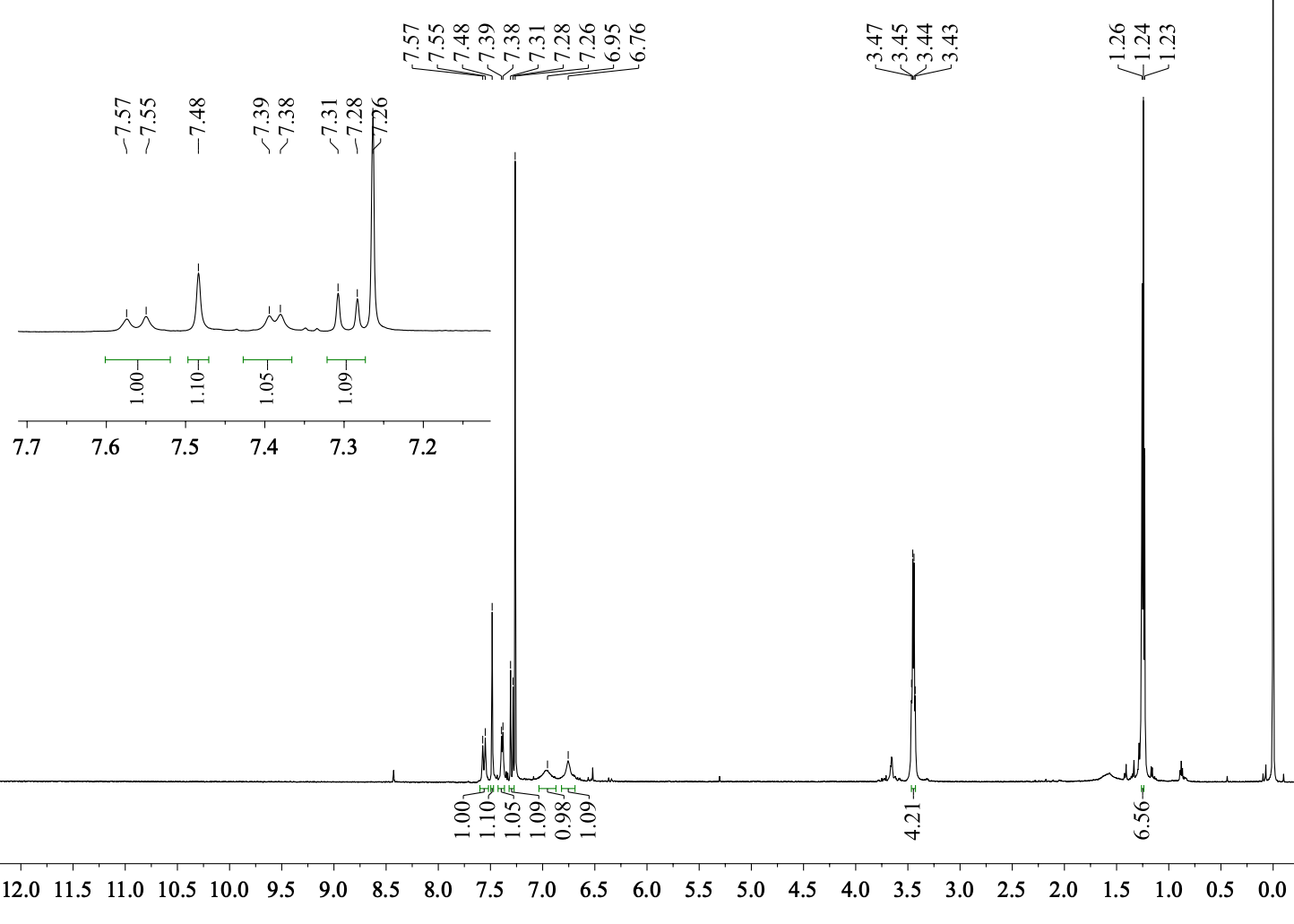


Fig S6. ^1^H NMR spectroscopy of compound **3**.


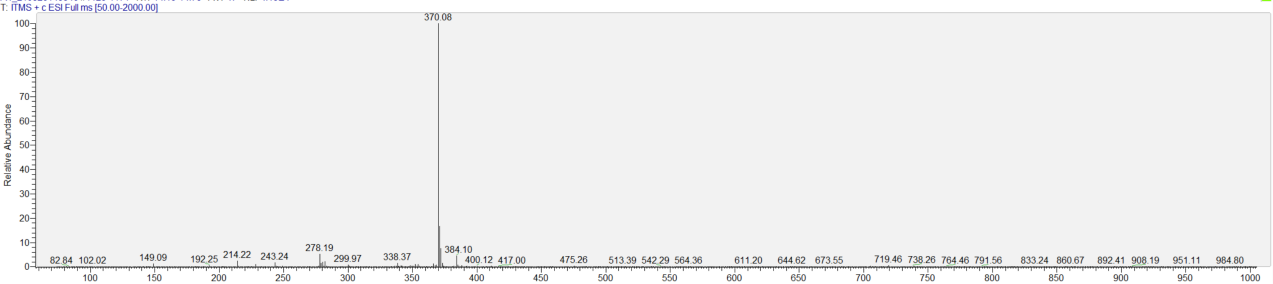


Fig S7. MS spectroscopy of compound **3**.


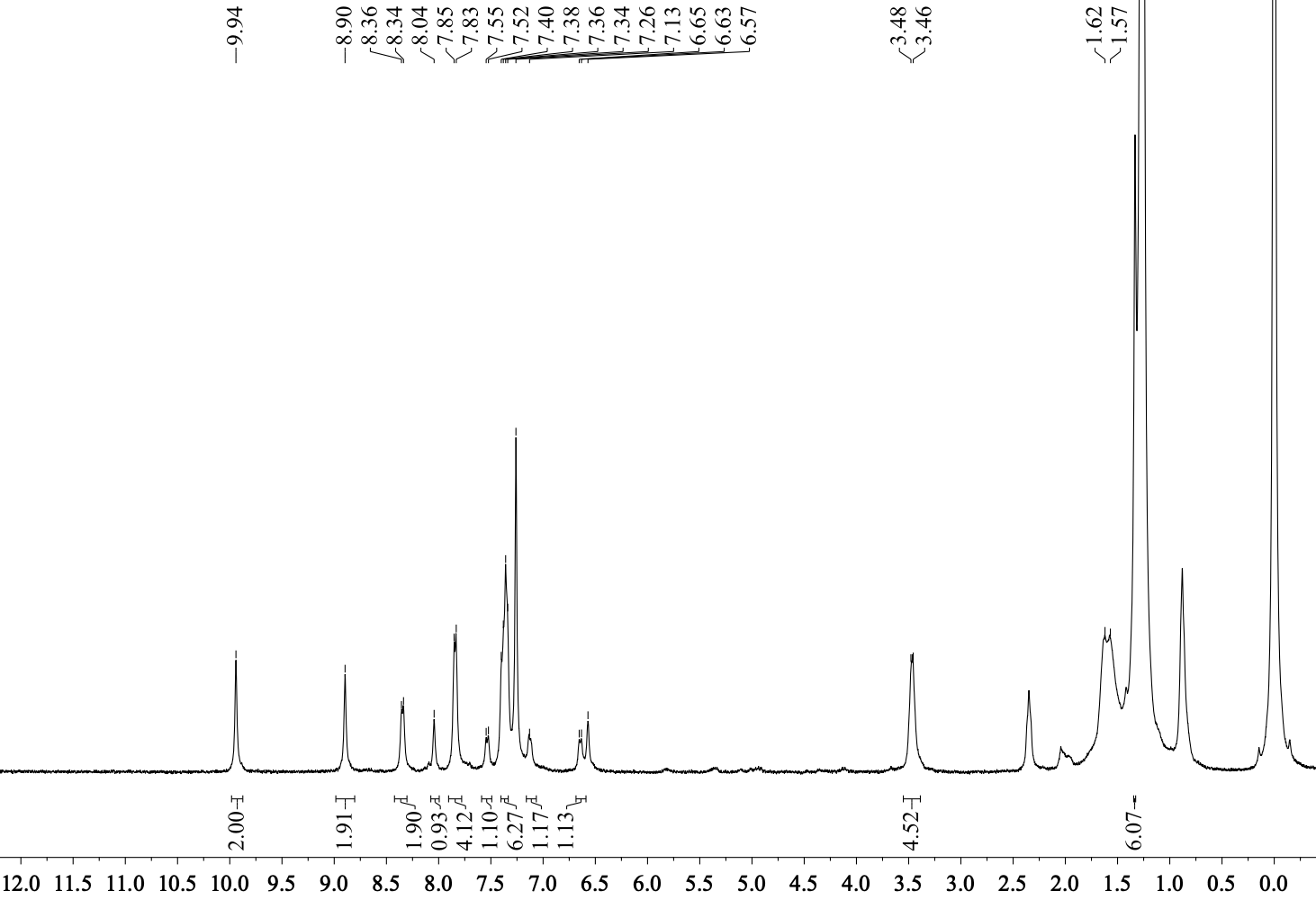


Fig S8. ^1^H NMR spectroscopy of compound **5**.

Fig S9. MALDI-TOF-MS measurement of compound **5**.


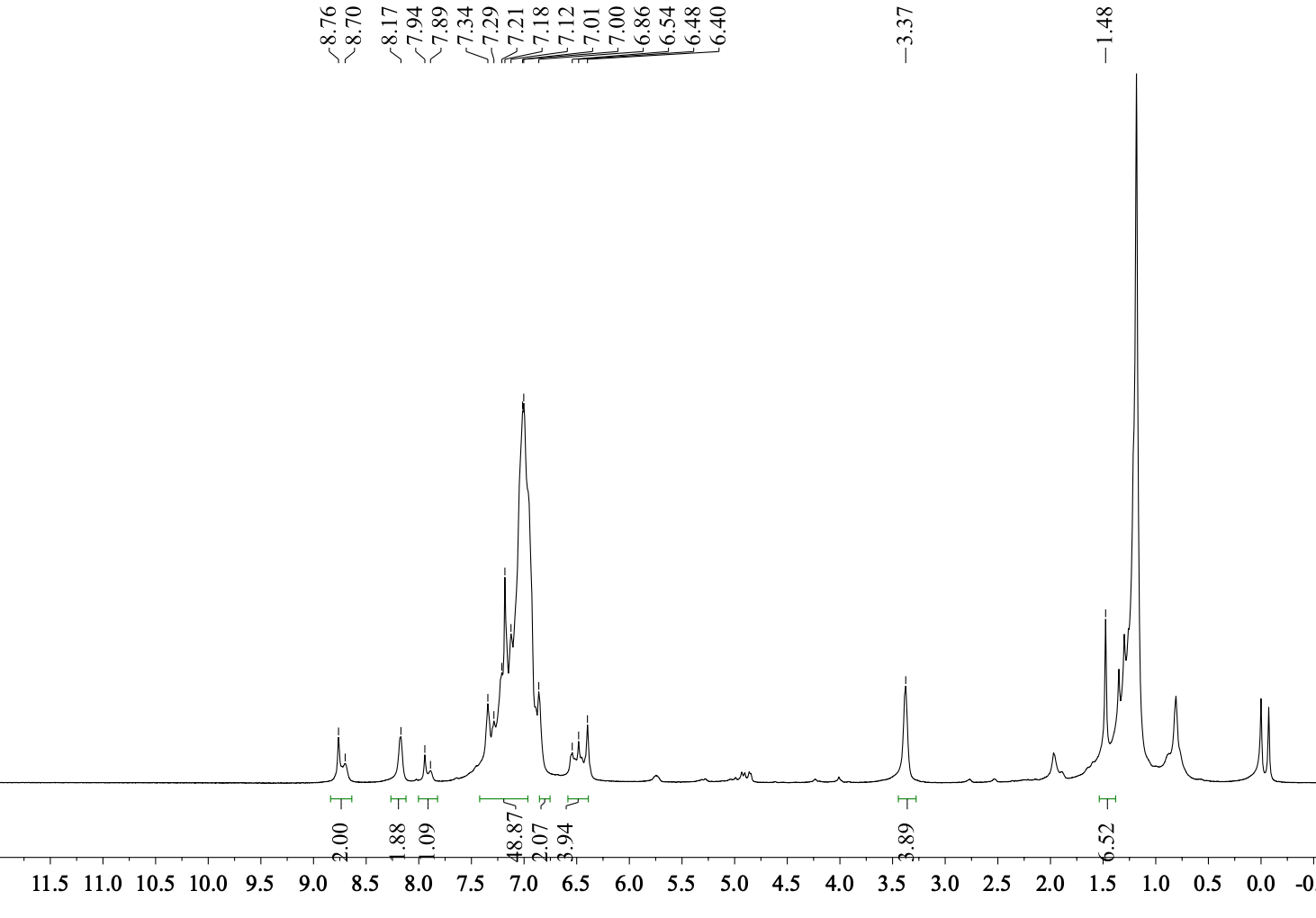


Fig S10. ^1^H NMR spectroscopy of compound **A1**.


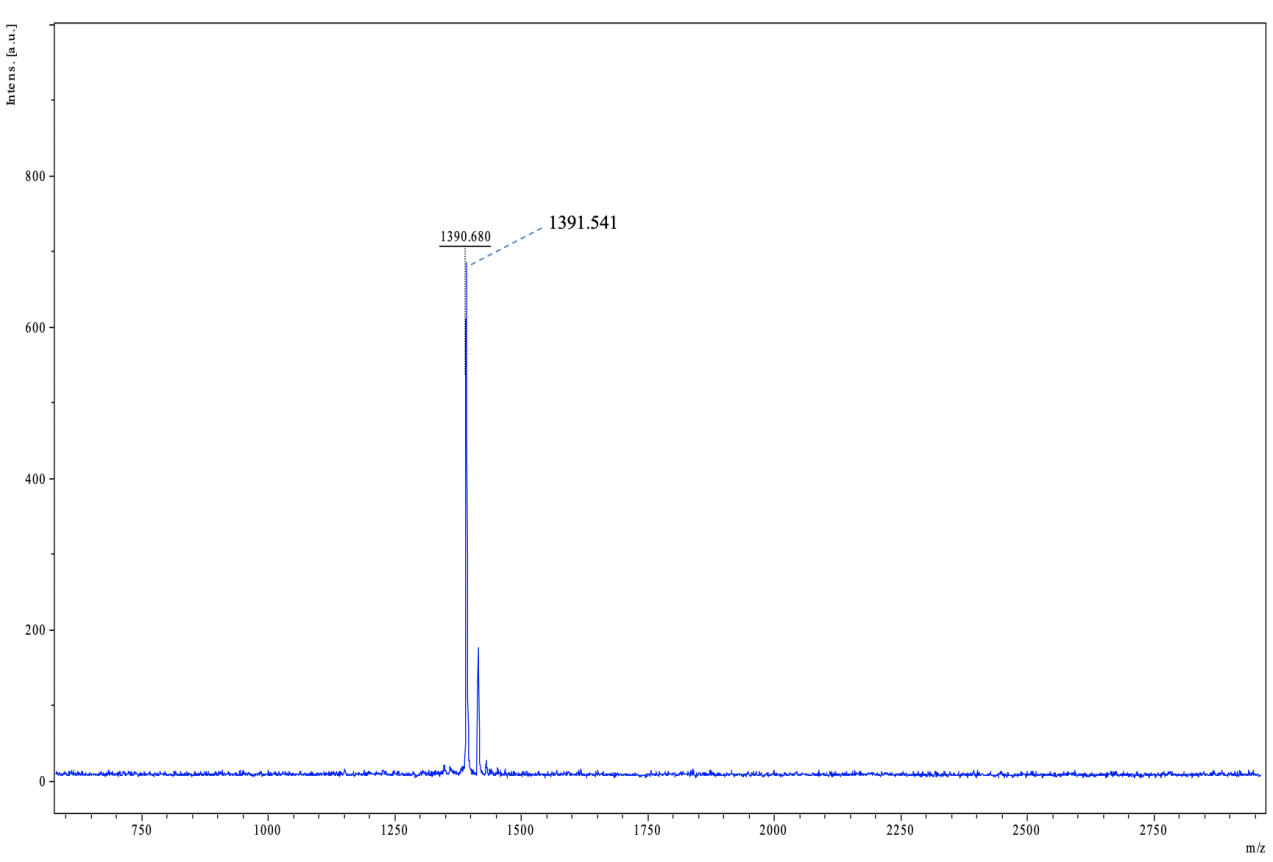


Fig S11. MALDI-TOF-MS measurement of compound **A1**.

**Preparation of A1** **NPs**

We prepared A1 NPs via the nanoprecipitation method. Specifically, 1mg A1 was mixed with 60mg f98 and the mixture was dissolved in 2ml DCM. Then, the mixture was oven-dried over-nigh at room temperature. After leaving overnight, the prepared A1 NPs were dissolved in DI water (3ml) and solutions were filtrated with 220 nm microfiltration membranes. Finally, the A1 NPs were stored at 4℃ in a refrigerator.

**Characterization of A1 NPs**

Dynamic light scattering (DLS) was used to characterize the size of the A1 NPs in aqueous solution. Transmission electron microscopy (TEM) images were captured to investigate the exact morphology of the A1 NPs.

Absorbance spectra of the A1 NPs were recorded on a Shimadzu UV-2600 UV-vis spectrophotometer. The emission spectra and absolute fluorescence quantum yields (QY) were measured by the FLS980 (Edinburgh Instruments). The fluorescence signal intensities were measured in water at various concentrations (10 ug/ml, 20 ug/ml, 30 ug/ml, 40 ug/ml and 50 ug/ml) and the linearity between fluorescent intensity and concentration was varified using a linear fit.

Photothermal properties tests: A1 NPs were diluted to different concentrations and then exposed to laser irradiation (808 nm, 0.8 W/cm^2^) for 5 min or A1 NPs (330ug/ml) were subjected to different laser powers for 5 min. The temperature profile of the A1 NPs solution was recorded with a Fluke Ti27 Infrared Camera. The photostability of A1 NPs was evaluated by monitoring the real-time temperature upon 808 nm laser irradiation over five cycles of heating/cooling processes.

For calculation of the photothermal conversion efficiency (η) under 808 nm laser irradiation, correlation equations were applied according to the previous report:

$\eta=\frac{hs(T_{\max}-T_{\mathrm{maxs}})}{I(1-{10}^{-A808})}$ (1)

$\theta=\frac{T-T_{\mathrm{sur}}}{T_{max}-T_{sur}}$ (2)

$T=-\tau_{s}In\theta$ (3) $\tau_{s}=\frac{\sum_{i} m_{i}C_{p,i}}{hs}$ (4)

Where I is the laser power, A^808^ is the absorbance of the A1 NPs at 808 nm, T_max_ and T_maxs_ are the maximum temperatures (°C) of A1 NPs and PBS upon laser irradiation, Where T is random temperature, and T_sur_ is the initial temperature, respectively. In order to get the hs that represent a dimensionless driving force temperature, θ is introduced: In this work, I=0.8, A808=0.486, T_max_-T_maxs=_80.1-48.4=31.7. m=1 g, C= 4.2 J g^-1^ °C, so$\sum_{i} m_{i}C_{p,i}$ =4.2×10^3^ [J/kg·℃]. The hs value was determined to be 9.33mW/°C and η value was 55.3%.

ROS generation of the A1 NPs: DCFH was used to detect the ROS generated by the A1 NPs. A strong green fluorescent substance DCF could be formed though DCFH reacting with ROS. The A1 NPs were dissolved in DCFH until OD value reached about 0.07. First, the fluorescence was recorded without NIR laser irradiation. Then, the A1 NPs were illuminated using an 808 nm laser (0.1W/cm^2^), and the fluorescence of DCFH was monitored at different post-irradiation times (1, 2, 3, 4, and 5 min).

**Cell Culture and In vitro cell experiments**

The luciferase-expressing 4T1 (4T1-Luc) tumor cells (Mall North na chuanglian biological Technology Co., LTD, China) grown in DMEM Medium (Gibco) containing 10% fetal calf serum (Life Technologies) and 1% penicillin/streptomycin solution (HyClone) and then were cultured at 37 °C under 5% CO_2_ and 95% air in a humidified incubator. Biocompatibility and killing ability of the A1 NPs against 4T1cancer cells were assessed with Cell Counting Kit-8 assay (CCK-8, Dojindo, Japan). 4T1-Luc cells were seeded into 96-well plates with 100 ul complete medium (n=3 per concentration) each well. When the degree of cell confluency reached 80%, the complete medium was discarded and 100ul medium containing different concentrations of A1 NPs solution was added. Then, the 96-well plate was placed in an incubator for 6 hours. After that, 10 ul of CCK-8 solution was added to each well and incubated for 2-4 h until OD value of control well reached 1.2. The cell viability (%) was calculated by [(Asample-Ablank)/ Acontrol-Ablank)] ×100%.

**Animal model**

Female Balb/c mice (6-7 weeks old) were purchased from Vital River (Beijing, China). The fur around the tumor site was shaved one day prior to injection of tumor cells. Orthotopic tumor model of breast cancer was constructed by direct 4rd pair of mammary fat pads injection of 1×10^6^ 4T1-Luc cells (75ul), and Bioluminescence imaging (BLI) was carried out using the IVIS Lumina II BLI system (Caliper, Hopkinton, MA, USA) to observe tumor growth and lymph nodes metastasis. Bioluminescence signals could be identified at the axillary lymph nodes and sciatic lymph nodes at day 21-28. Mice were sacrificed when tumor volume reached to 2000 mm^3^. The size of the tumor was measured by vernier caliper and volume was calculated according to the following formulation: V = (length × width^2^) × 0.52.

**In vivo NIR-II fluorescence imaging**

To investigate the metabolic status of probes in tumors, NIR-II fluorescent imaging was performed in 3 orthotopic bearing mice. First, they were injected with A1 NPs (165ug/ml, 100ul) through the tail vein. Then, we used 808 nm laser was as excitation source and InGaAs SWIR camera (NIR vana 640; Princeton Instruments) to capture NIR-II fluorescence images mice at different time points. The specified parameters were: the InGaAs camera was cooled to -80 °C, the exposure time was set to 500 ms, and 1,100 nm long-pass filter (Thorlabs) was used and the excitation laser power was 30–35mW/cm^2^. During in vivo NIR-II fluorescence imaging, the mice were anesthetized by isoflurane (2%). The tumor-to-background ratio (TRB) were calculated as: (mean fluorescent signal intensity of tumor) / (mean fluorescent signal intensity of mirror areas).

**In vivo** **NIR-II fluorescence imaging-guided breast tumor resection**

A1 NPs solution (165ug/ml, 100ul) was injected intravenously 24h prior to surgery. First, before tumor resection, NIR-II fluorescence imaging was used to delineate the scope of the tumor. Then surgeon relied on inspection and palpation to remove tumor. After the surgeon subjectively felt that he had completely removed the tumor, an 808 nm laser was again used to scan the tumor cavity. Once detected the presence of fluorescent signal left in tumor cavity, surgeon removed them under the guidance of fluorescence imaging. Until no fluorescence signals were detected in the surgical area, the skin incision was sutured.

**In vivo NIR-II fluorescence imaging-guided metastasized lymph nodes resection**

After sciatic lymph nodes and axillary lymph nodes were observed bioluminescence signals, mice were injected intravenously A1 NPs (165ug/ml, 100ul). At 24 h, NIR-II fluorescence imaging was carried out and lesion with fluorescence signal was removed. Afterwards, the mice were sacrificed, and the rest of the enlarged lymph nodes were harvested. All the ex vivo lymph nodes was performed BLI, FLI and H&E staining.

**In vivo anticancer studies**

When the diameter of the tumor reached 5-6mm (At the 7th day after injection of tumor cells), The 4T1 tumor-bearing female BALB/c mice were randomly divided into four groups (n=6): PBS group, NPs group, Laser group, and NPs+Laser group. The experimental group is NPs+ Laser group, in which A1 NPs solution was administrated via tail vein for each mouse. After 24h, 808nm laser irradiation (1W/cm^2^) was conducted for mice for 10 min. The other three control groups were set at the same time, such as PBS group (only injections of 100ul PBS), NPs group (only injections of 100ul A1 NPs solution) and laser group (without injections of A1 NPs solution, only 808nm laser irradiation for 10min). Tumor temperature was recorded every 2 minutes during irradiation in NPs+Laser group and Laser group by Fluke Ti27 Infrared Camera. The body weight and tumor size of each mouse were recorded every other day. At day 7, a subset of mice (n=3 in each group) were euthanized and the tumor-draining lymph node and spleen were removed for flow cytometry analysis. At day 18, the remaining mice (n=3 in each group) were sacrificed and tumors were harvested for histopathological evaluations and tumor weight measurement.

**Adjuvant Phototherapy Procedure**

Tumor-bearing mice were i.v. injected 100ul A1 NPs 24 h prior surgery. The residual tumor model was constructed by intentionally remaining part of the tumor in the surgical cavity. The surgical cavity was irradiated with a 808nm laser for 10 minutes, and the residual tumor temperature was recorded every two minutes. BLI was performed to monitor local recurrence and distant metastasis after surgery. Mice were killed at day 40, the lungs and tumor draining lymph node were harvested. Lymph node metastasis was by evaluated by BLI. Lung metastasis was examined with the naked eye and H&E staining.

**H&E Staining and Tunel staining**

Resected tumor tissues and lymph nodes was fixed in 4% paraformaldehyde solution and embedded with paraffin. Tumors and lymph nodes specimens were cut into 3–4mm pieces, then the sections were stained with hematoxylin and eosin. TUNEL staining was performed using Fluorescein (FITC) Tunel Cell Apoptosis Detection Kit. All Pictures of stained sections were captured using a digital scanner (Pannoramic MIDI, 3DHistech).

**Frozen sections**

Tumor tissues harvested from mice were embedded with OCT and then made into 5 μm frozen sections. We used home-made NIR-II fluorescence microscope to image frozen sections.

**ICD biomarkers detection**

To determine phototherapy-induced ICD of the 4T1-Luc tumor cells in vitro, calreticulin (CRT), high-mobility group protein B1 (HMGB1) and heat shock proteins (HSP) 70 were examined via immunofluorescence staining. 4T1-Luc cells were seeded 20mm small confocal dishes and cultured to 80% confluency. They were allocated into 2 groups: A1+Laser group and PBS group. For the A1+Laser group, then the culture media were replaced with fresh media containing A1 NPs and the cells were incubated for 6 h. After 808nm laser light irritation (1W/cm^2^, 5 min), the cells were cultured overnight. Subsequently, the media were replaced by the PBS. For the PBS group, no A1 and laser irritation. For different makers staining, the cells were fixed, permeabilized and then blocked, then incubated with different antibodies (ab92516, ab181606), followed by a further incubation at room temperature for 1h with a goat secondary antibody to Rabbit IgG (Alexa Fluor® 488) (ab150081). The nuclear counter stain is DAPI (blue). The cell nuclei were labelled with DAPI. PBS washes were performed after each step for 5 minutes. At last, the stained cells were imaged by a CLSM.

**Intracellular ROS detection**

Reactive oxygen species (ROS) generation ability of the intracellular A1 NPs was detected by DCFH-DA. Specifically, 4T1-Luc cells was cultured in confocal dish until cell confluency reached 80%, and A1 NPs solution diluted with DMEM was added into confocal dish. After 6 h post-incubation, the cells were exposed to an 808 nm laser (1 W/cm^2^) for 5 min. After overnight, the medium was discarded and replaced with 1ml fresh medium containing the DCHF-DA (1ul). Then 30 min later, the cells were washed three times with PBS, and the 1ml PBS was added to the dishes. Subsequently, LEICA DMIL LED was employed to detect the ROS generation ability of the A1 NPs.

**Flow cytometric analysis**

The sources of antibodies for flow cytometric analysis were indicated where they appeared in the text. Lymph node and spleen were harvested and minced into small pieces using scissors. A single-cell suspension was prepared using cell strainer and removed of red blood cells (RBC) using the RBC lysis buffer. The obtained cells were stored in the cell staining buffer at 4 °C. For blood samples, blood was centrifuged for 15 min at 1500 rpm at 4 °C to remove serum. Blood cells were incubated with RBC lysis buffer and then centrifuged for 15 min at 1500 rpm to collect lymphocytes. For dendritic cell maturation, cells from lymph node were stained with anti-CD45-vFluor450, anti-CD11c-FITC, anti-CD80-PE, anti-CD86 PerCP-Cy5.5 antibodies according to the manufacturer’s protocols, and anti-CD86-PE CD11c+CD80+CD86+ cells were defined as mature dendritic cells. For cytotoxic T lymphocytes (CTLs) in spleen tissue, cells were stained with anti-CD3-FITC, anti-CD4PerCP-Cy5.5, anti-CD8-PE, anti-CD45-vFluor450 antibodies, and the CD3+CD4-CD8+ cells were CTLs. Cytokine concentrations in the serum were analyzed with a CBA Mouse Inflammation Kits according to the manufacturer’s instructions.

**Biosafety evaluation**

At the 7th day after treatments, the main organs of the mice in every groups were exercised for H&E staining. Blood samples of the mice were collected for blood biochemical analysis. Six hematological indexes of the mice, including alanine aminotransferase (ALT), blood urea nitrogen (BUN), lactate dehydrogenase (LDH), aspartate transaminase (AST) and alkaline phosphatase (ALP), were analyzed using a Chemray 800 Automated Chemistry Analyzer (Shenzhen Rayto Life and Analytical Sciences).

**Statistical analysis**

Quantitative results were presented as mean ± standard deviation. Statistical significance was determined using an unpaired two-tailed Student t test with GraphPad Prism 8. (n.s. P > 0.05, *p < 0.05, **p < 0.01, ***p < 0.001).





Fig S12. a) The hydrodynamic particle diameter and b) the fluorescence intensity of A1NPs in PBS during14days (0h, 1d, 3d, 7d and 14d).


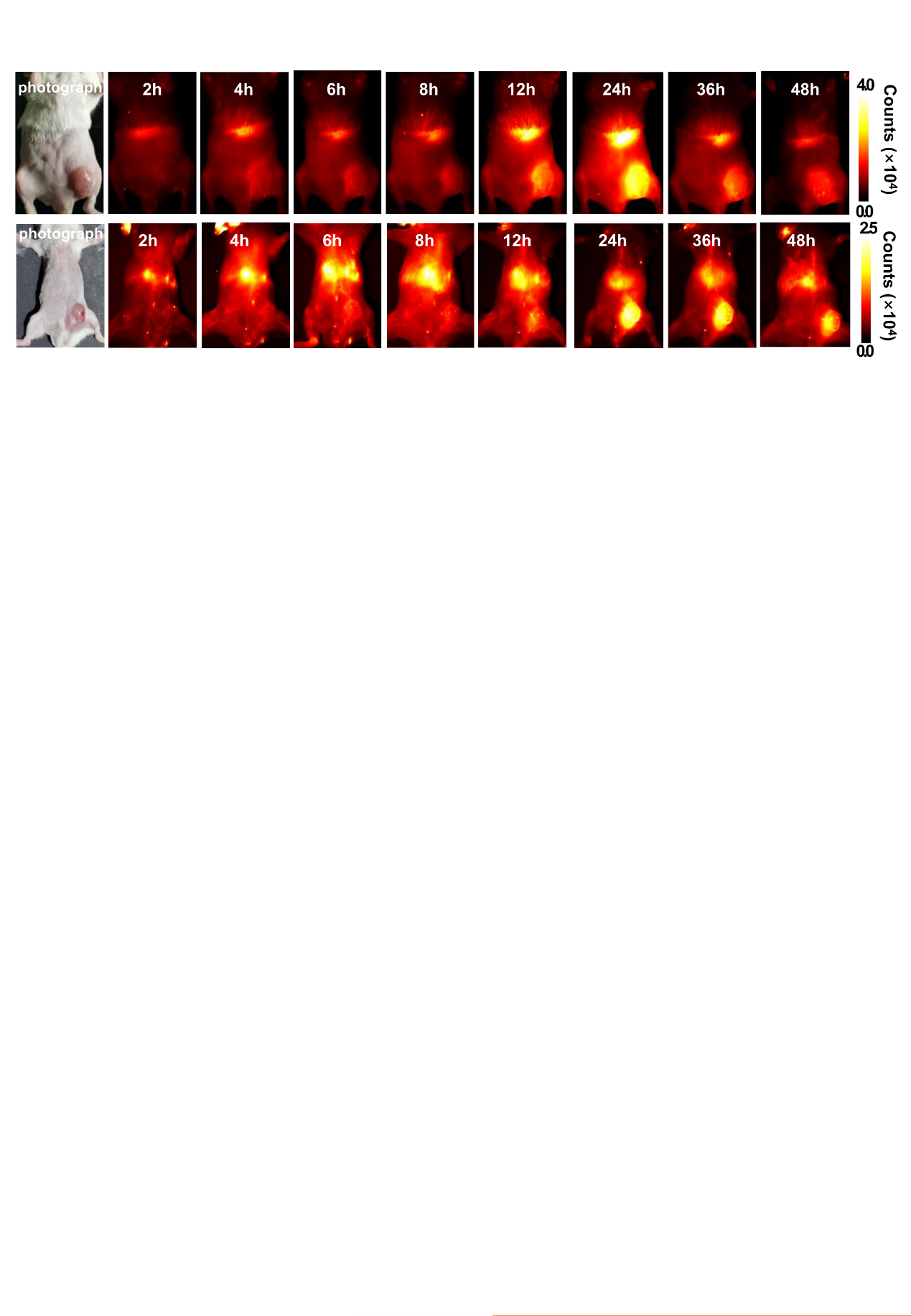


Fig S13**.** NIR-II fluorescence images of 4T1 tumor-bearing mice at various time points after tail-vein administration of the A1 NPs.


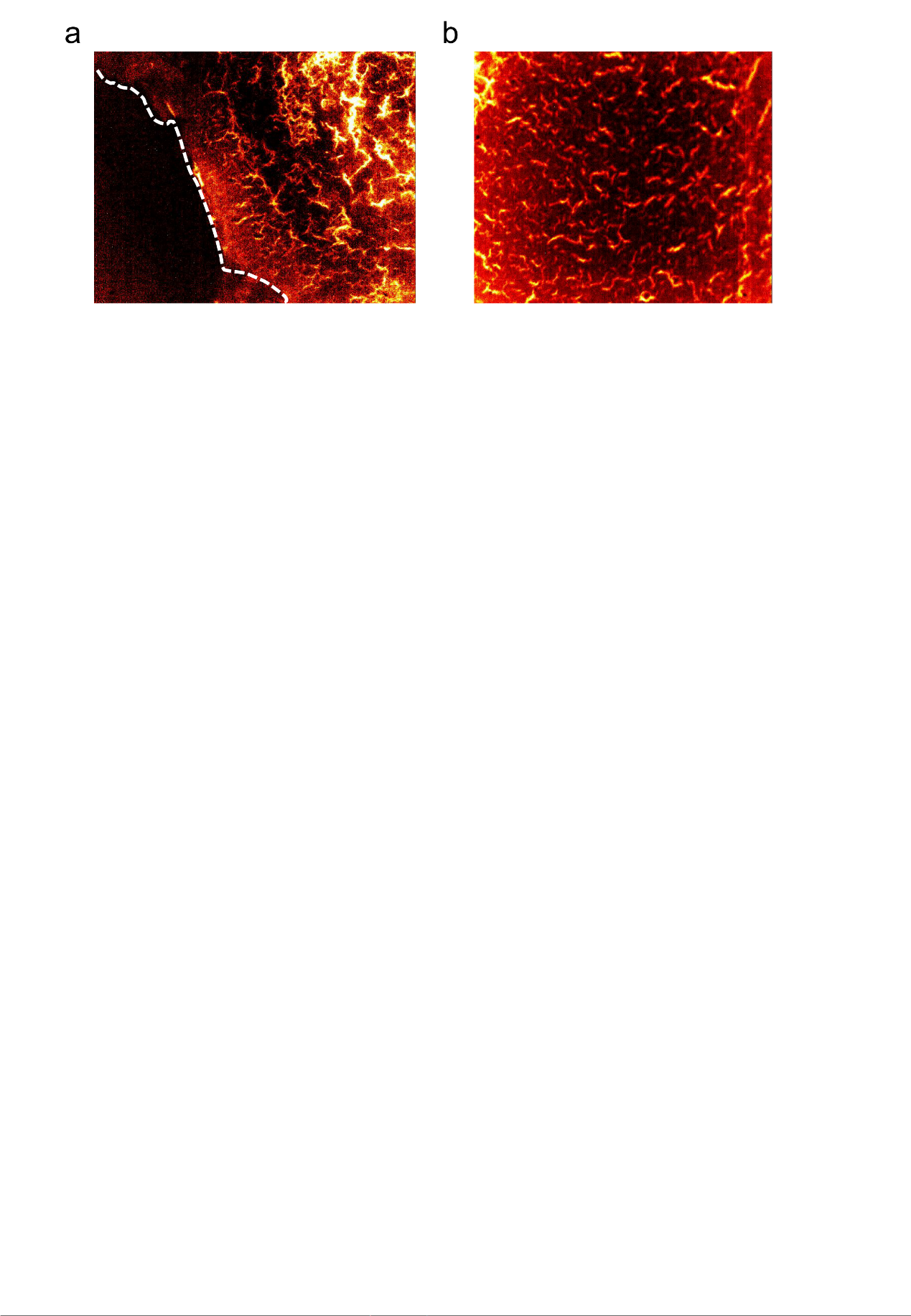


**Fig S14.** NIR-II fluorescence images of tumor frozen sections.


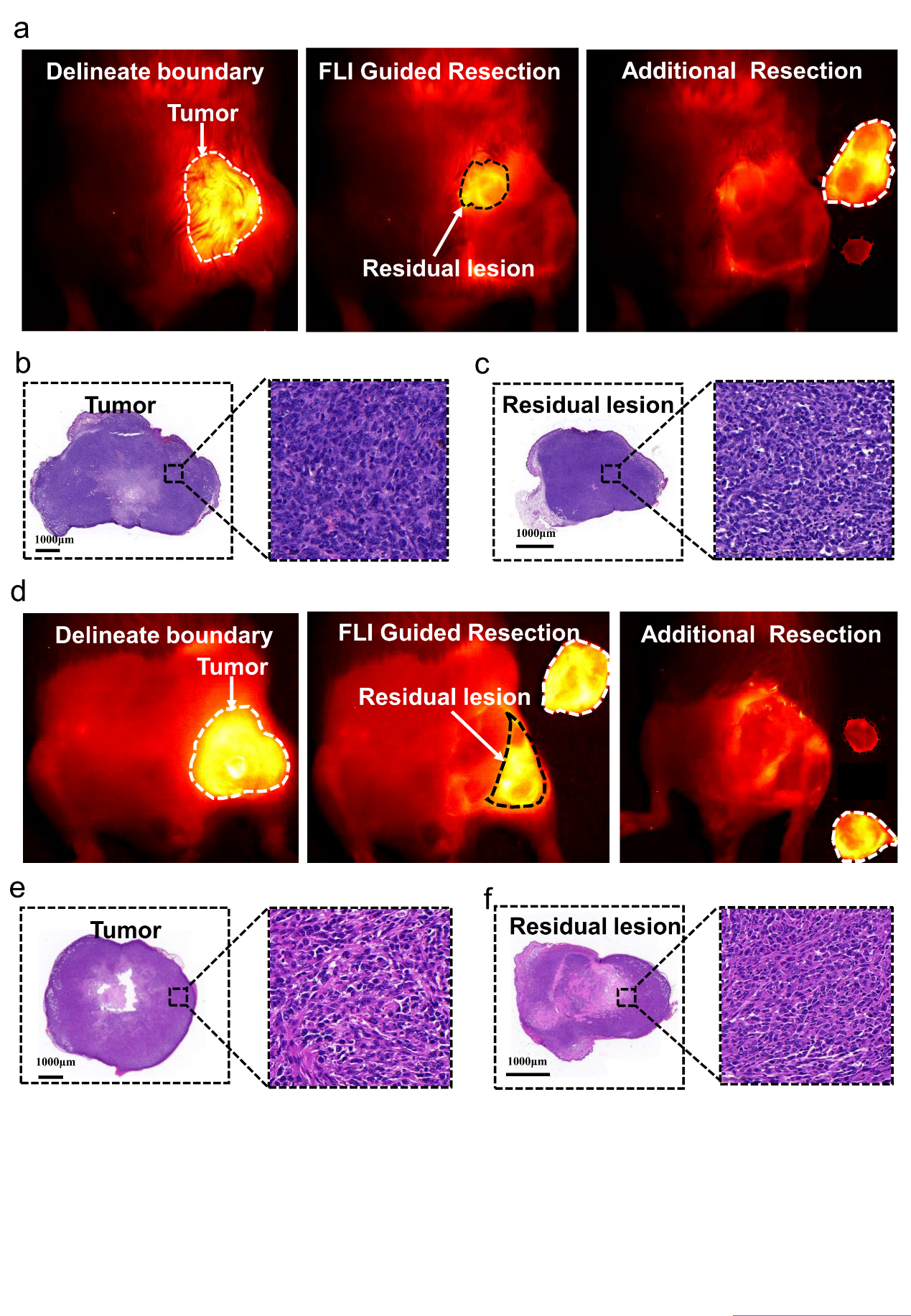


**Fig S15.** a, d) NIR-II images of orthotopic 4T1 breast tumor resection procedure. b, c, e, f) H&E staining images of excised tumor and residual lesion.


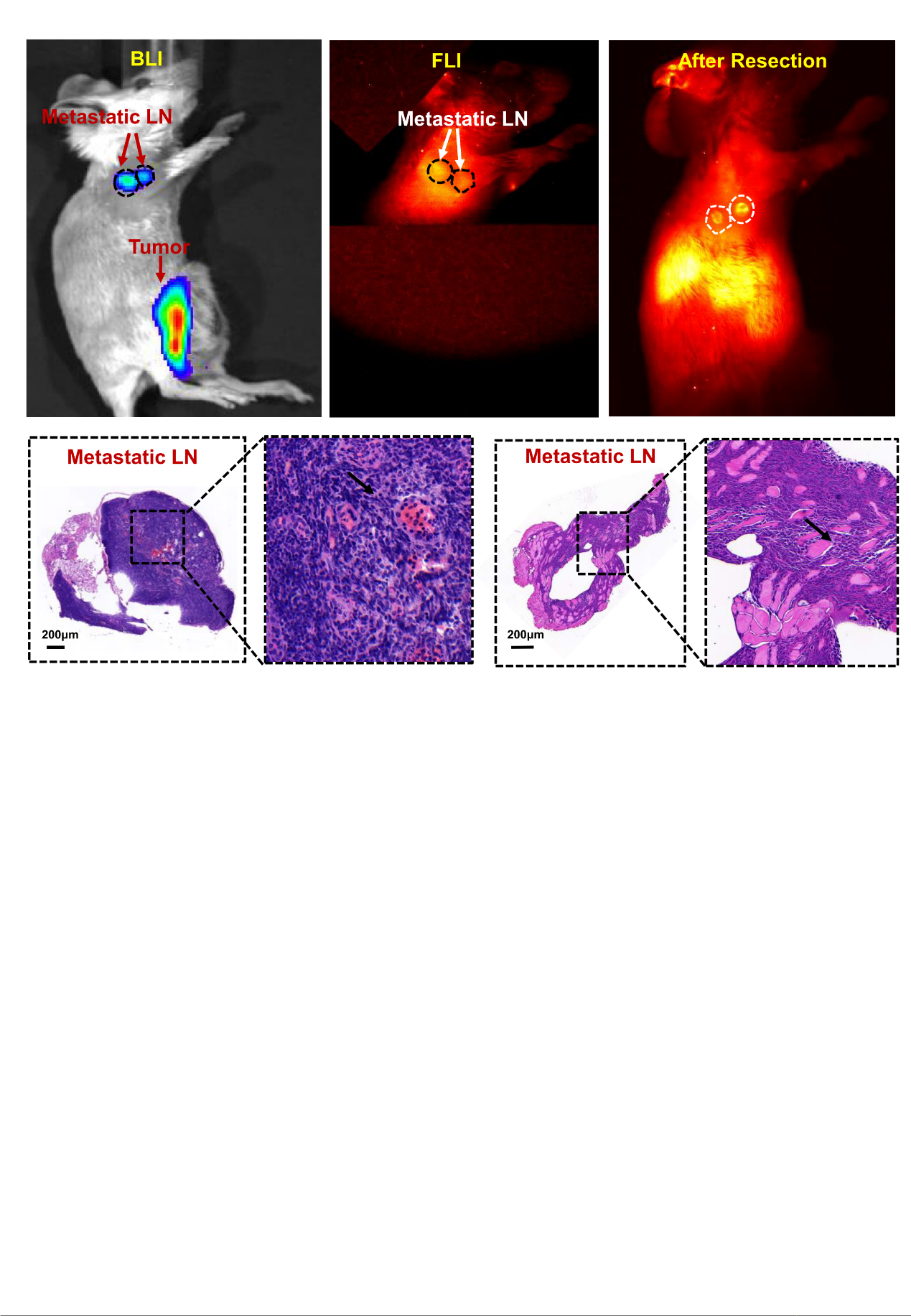


**Fig S16.** BLI images detected two positive sciatic lymph nodes in orthotopic breast cancer mouse. A clear fluorescent signal matched well with that of the bioluminescence imaging (BLI). Positive lymph nodes were resected under the NIR-II fluorescence imaging. Metastatic lymph nodes were confirmed by H&E staining.


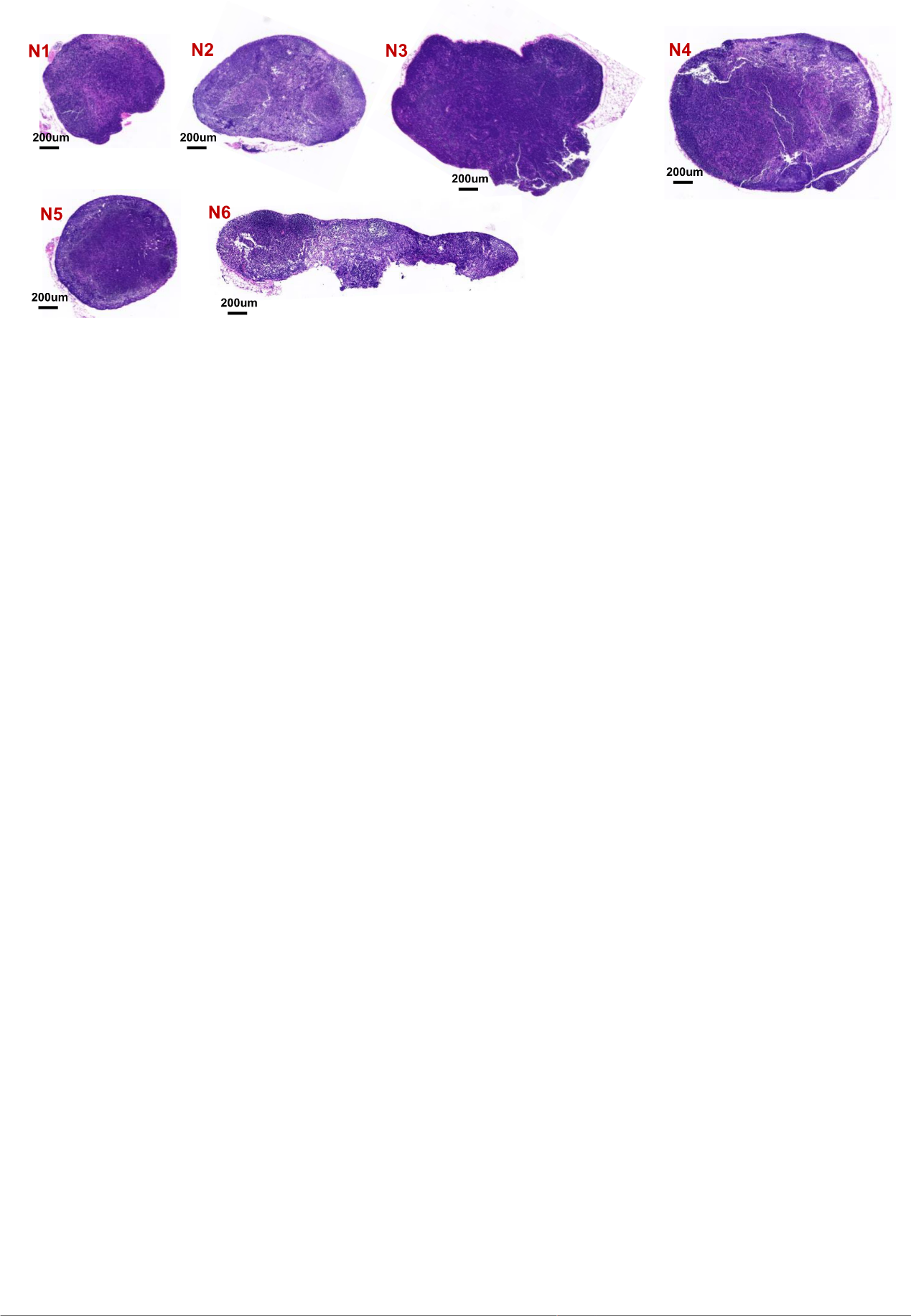


**Fig S17.** H&E staining of remaining enlarged lymph nodes in sciatic lymph node metastasis mice


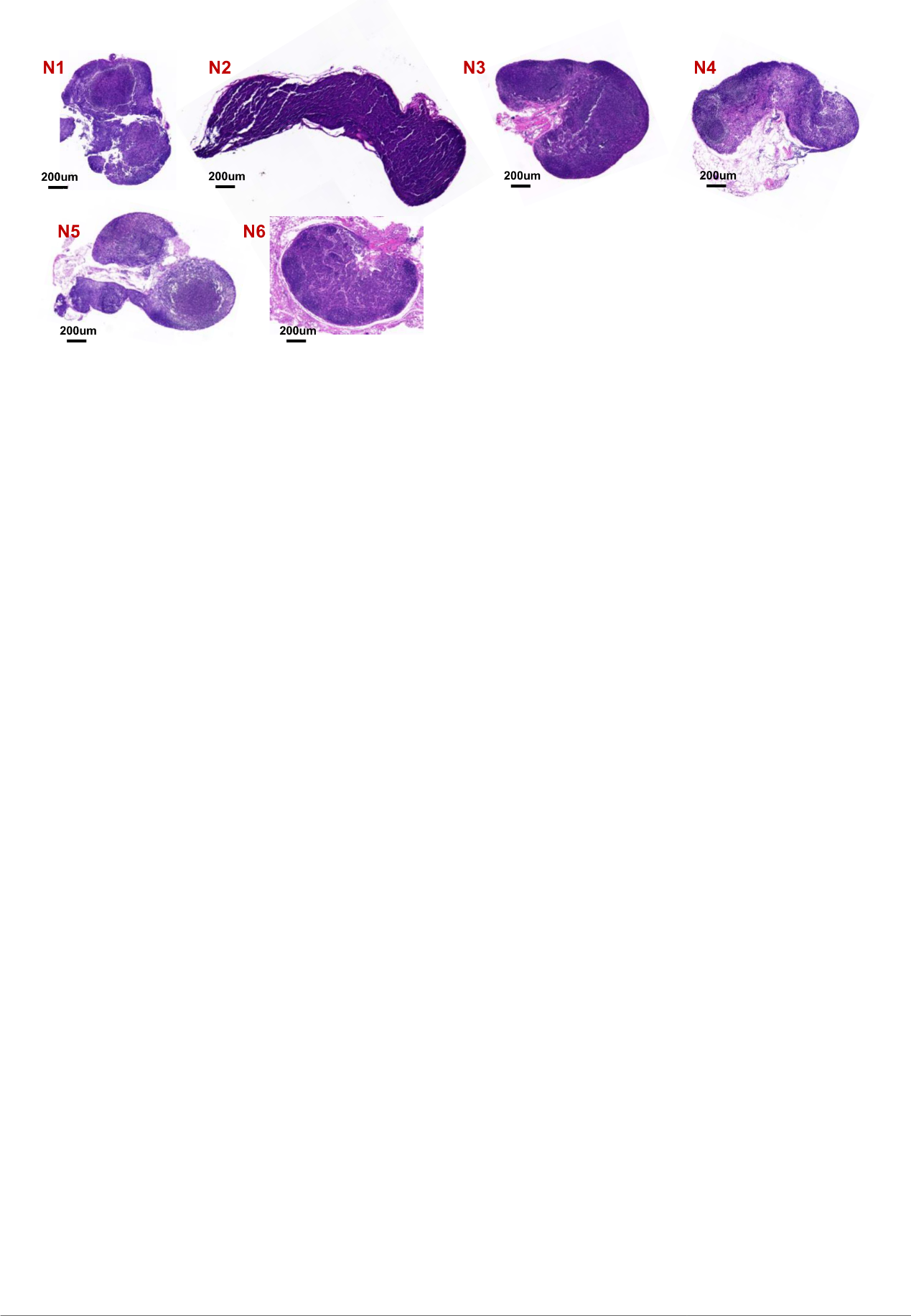


**Fig S18.** H&E staining of remaining enlarged lymph nodes in axillary lymph node metastasis mice.


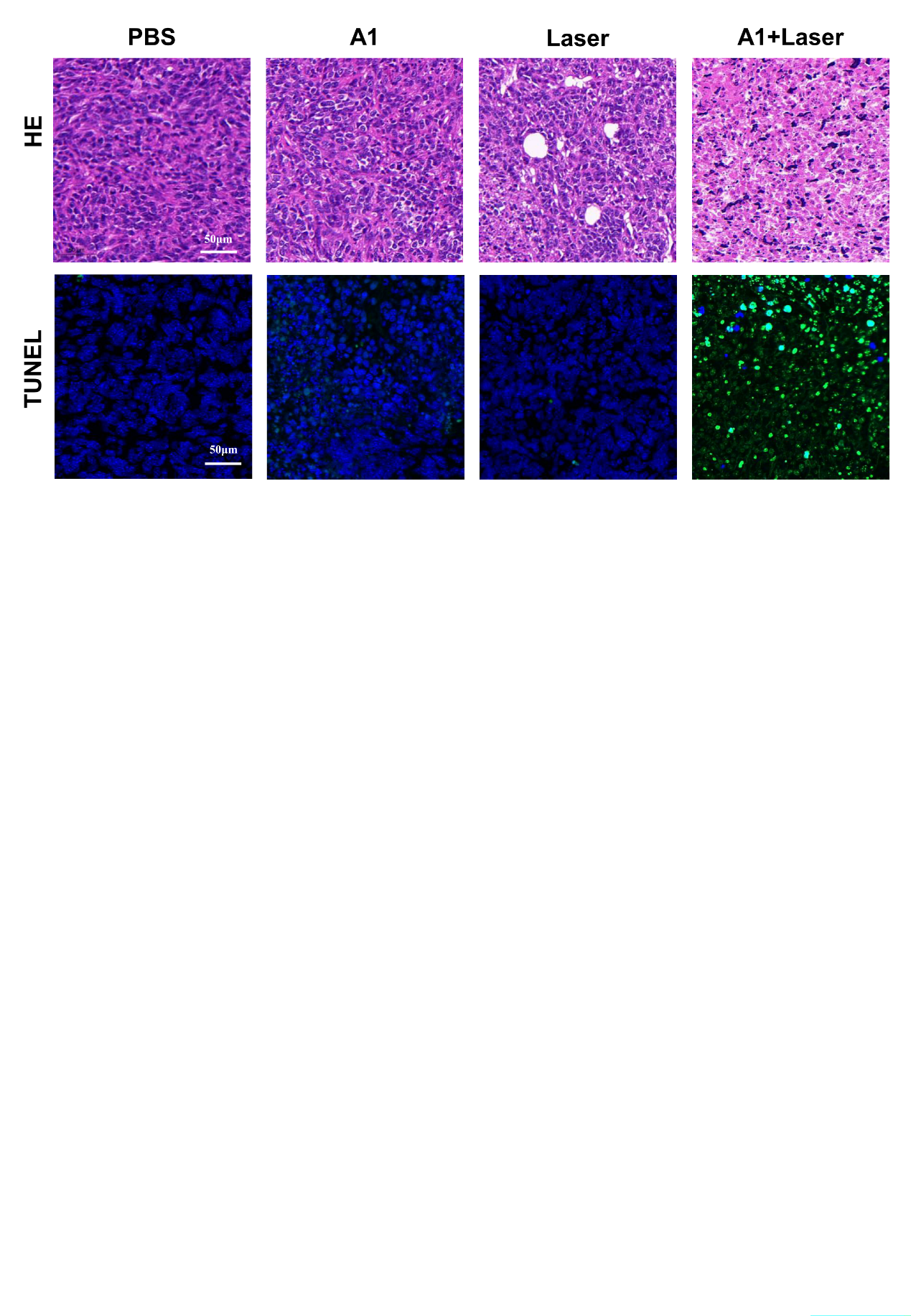


**Fig S19.** Representative H&E staining and CLSM images of tumors after TUNEL staining.

**
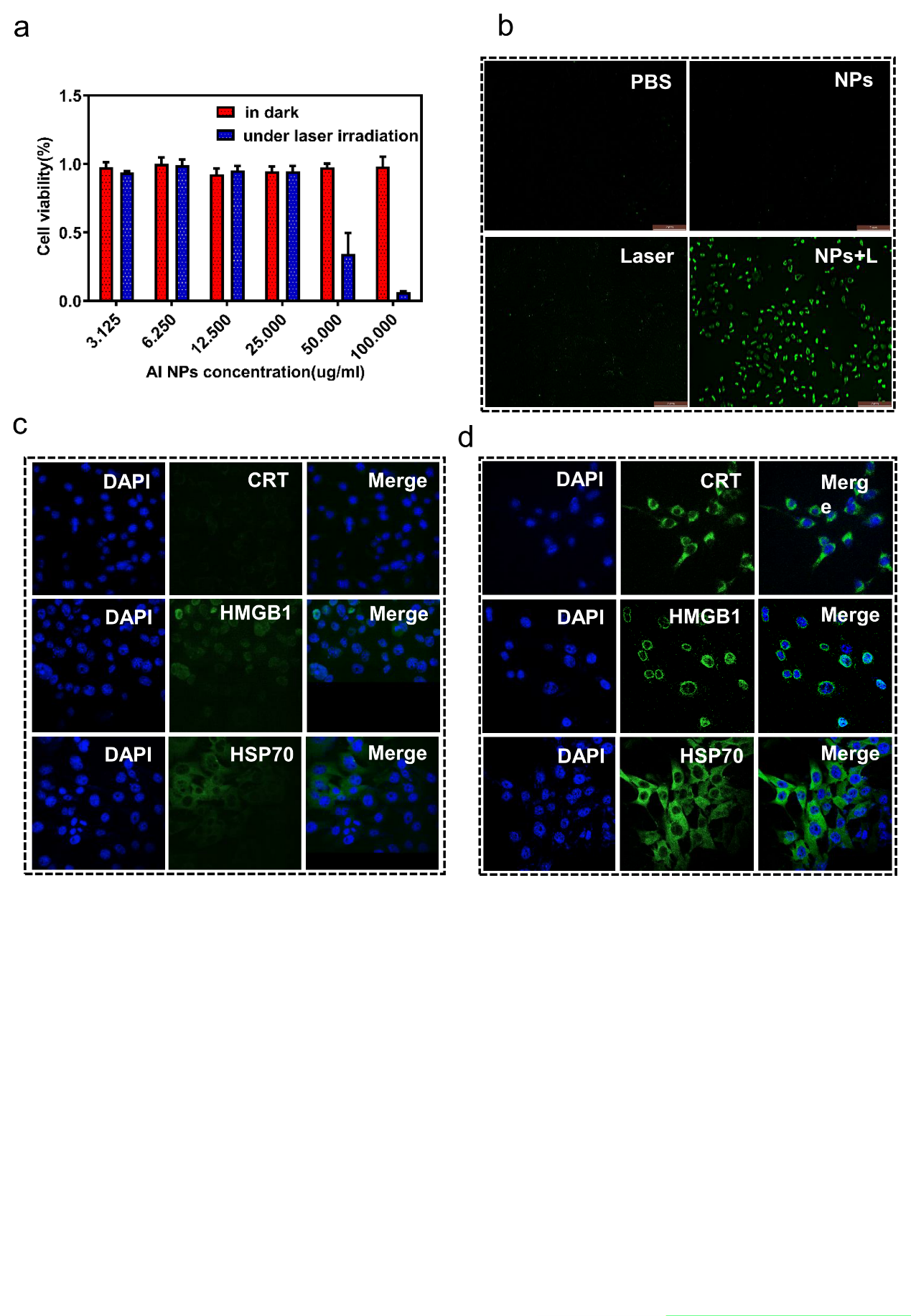
**

**Fig S20.** a) Cytotoxicity assays of different concentrations A1 NPs in the dark (incubation for 24 h) or under laser exposure for 5 min (808 nm, 1 W/cm^2^). b) ROS generation detection from 4T1-luc cells with different treatment using DCFH-DA. c) Immunofluorescence staining of CRT, HMGB1 and HSP 70 after 4T1-luc cells treated with PBS. d) Immunofluorescence staining of CRT, HMGB1 and HSP 70 after 4T1-luc cells treated with A1+Laser.


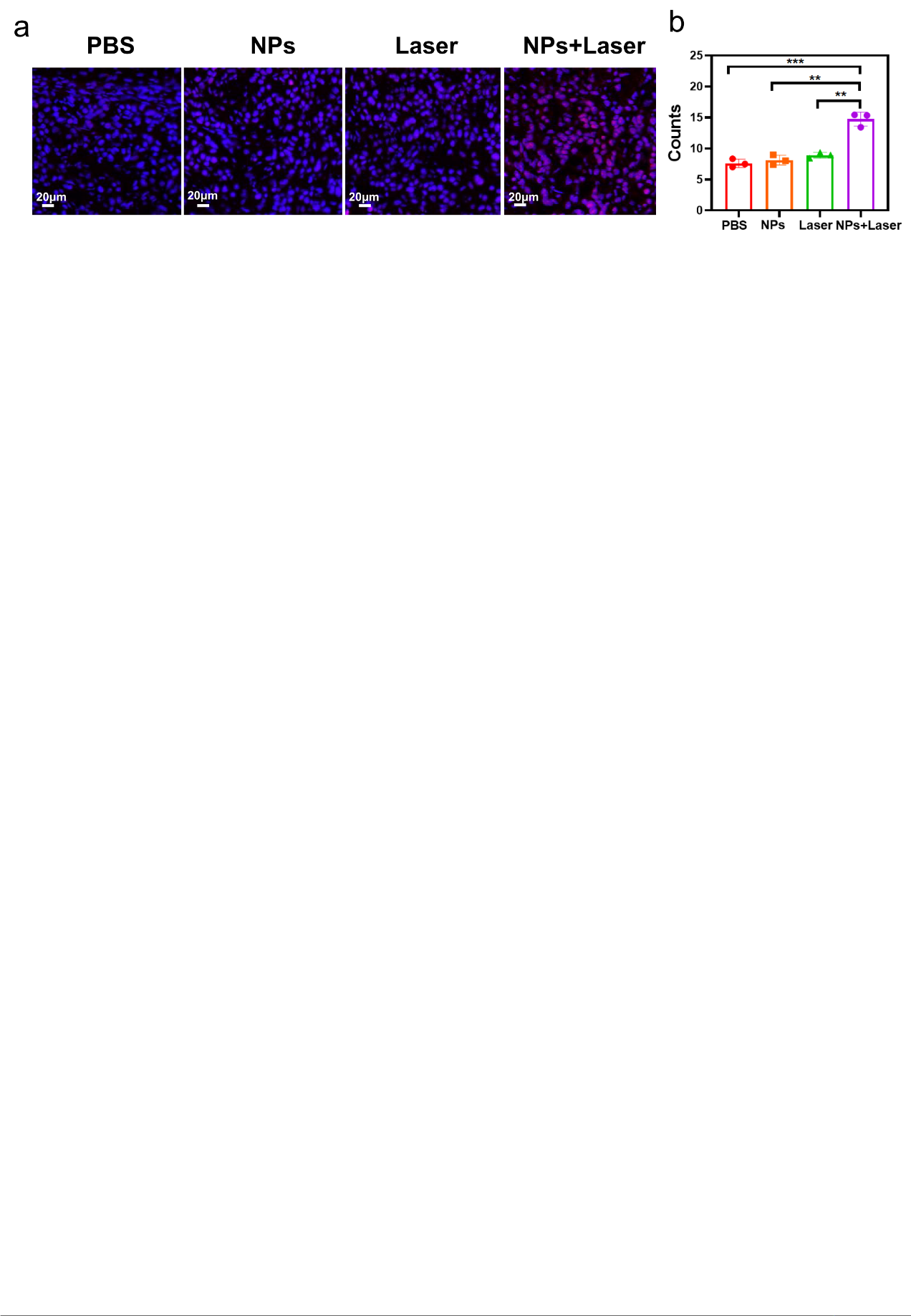


Fig S21. (a) Representative merge images include DAPI staining (blue) and ROS staining (red) of tumor tissue after the different treatments; (b) Fluorescence intensity analysis of ROS in different groups. (**P < 0.01, ***P <0.001).

**
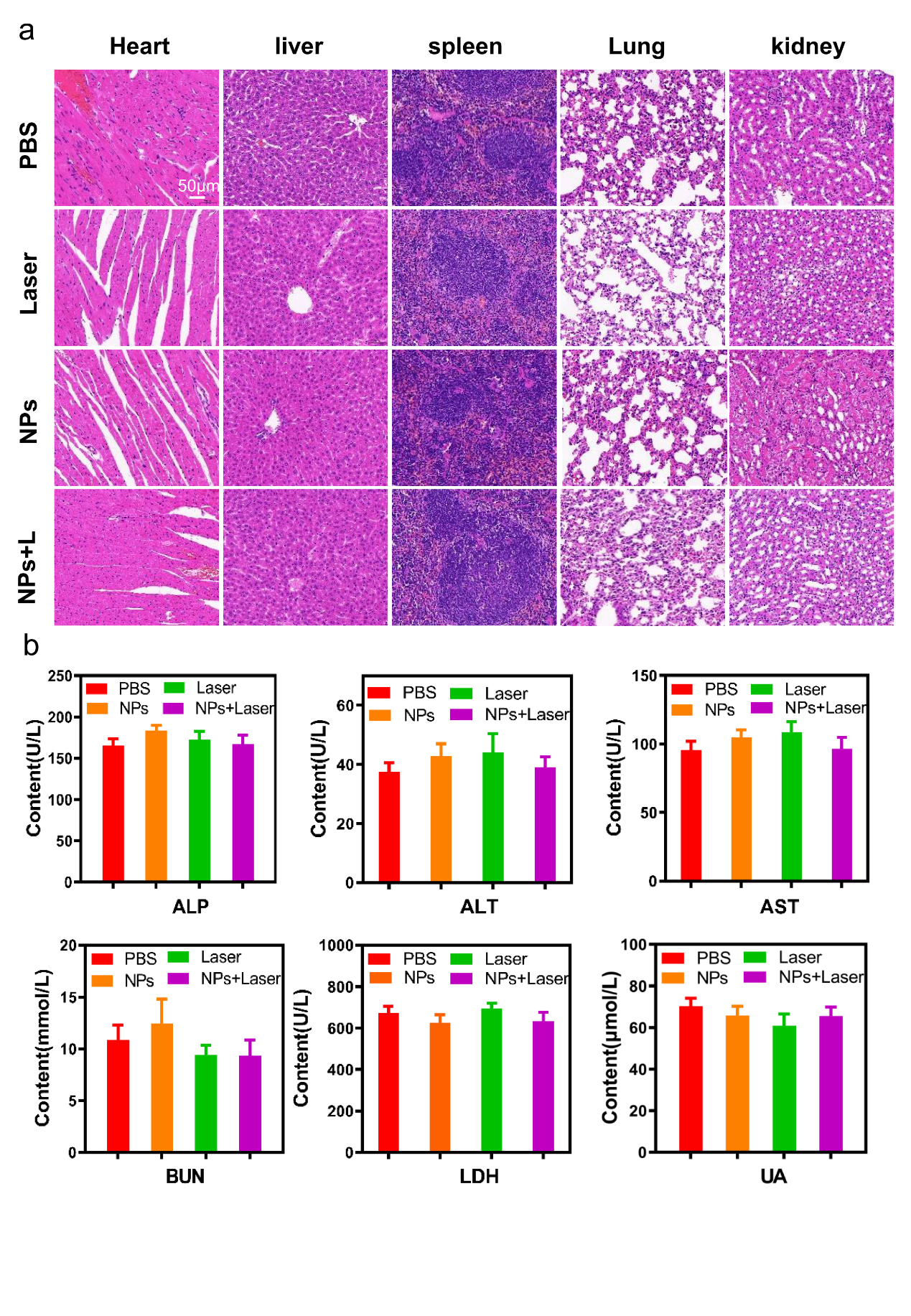
**

**Fig S22.** a) H&E staining of the main organs from the mice in different groups. b) Blood biochemistry analysis of the mice receiving different treatments.
